# Recent Progress in High Linearly Fused Polycyclic Conjugated Hydrocarbons (PCHs, *n* > 6) with Well‐Defined Structures

**DOI:** 10.1002/advs.201903766

**Published:** 2020-04-22

**Authors:** Wangqiao Chen, Fei Yu, Qun Xu, Guofu Zhou, Qichun Zhang

**Affiliations:** ^1^ Guangdong Provincial Key Laboratory of Optical Information Materials and Technology and Institute of Electronic Paper Displays National Center for International Research on Green Optoelectronics South China Academy of Advanced Optoelectronics South China Normal University Guangzhou 510006 P. R. China; ^2^ School of Materials Science and Engineering Nanyang Technological University 50 Nanyang Avenue Singapore 639798 Singapore; ^3^ College of Materials Science and Engineering Zhengzhou University Zhengzhou 450001 P. R. China

**Keywords:** antiaromaticity, higher acenes, organic field‐effect transistors, organic photoelectronic materials, polycyclic conjugated hydrocarbons (PCHs), single crystal packing

## Abstract

Although polycyclic conjugated hydrocarbons (PCHs) and their analogues have gained great progress in the fields of organic photoelectronic materials, the in‐depth study on present PCHs is still limited to hexacene or below because longer PCHs are insoluble, unstable, and tediously synthesized. Very recently, various strategies including on‐surface synthesis are developed to address these issues and many higher novel PCHs are constructed. Therefore, it is necessary to review these advances. Here, the recent synthetic approach, basic physicochemical properties, single‐crystal packing behaviors, and potential applications of the linearly fused PCHs (higher than hexacene), including acenes or π‐extended acenes with fused six‐membered benzenoid rings and other four‐membered, five‐membered or even seven‐membered and eight‐membered fused compounds, are summarized.

## Graphical abstract

Polycyclic conjugated hydrocarbons (PCHs) and their analogues have a wide range of applications in the fields of organic photoelectronic materials. This work provides a timely summary of recent advances in the latest synthetic routes to higher PCHs as well as their basic physicochemical properties, single crystal packing behaviors and potential applications.

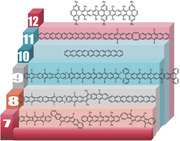

## 1 Introduction

Polycyclic Conjugated Hydrocarbons (PCHs) has attracted wide research interests in the past two decades due to their synthetic challenging and broad applications in organic electronics.^[^ ^1^ ^]^ Among all family members of PCHs, acenes, defined as linearly annulated systems containing three or more fused benzene rings and only possessing one electron sextet, are considered as one of the most important candidates for high‐performance organic field‐effect transistors (OFETs),^[^ ^2^ ^]^ organic light‐emitting diodes,^[^ ^3^ ^]^ etc. For example, 9,10‐di(naphthalen‐2‐yl)anthracene can be used as a promising luminescent material for a full‐color organic electroluminescent device to produce an effective and stable blue light,^[^ ^3a^ ^]^ while the single crystal of 5,6,11,12‐tetraphenyl‐substituted tetraacene (rubrene) has been demonstrated to exhibit a hole mobility up to 40 cm^2^ V^−1^ s^−1^.^[^ ^4^ ^]^ When the number of the linearly fused benzenoid rings increases, the tedious synthesis to construct them and their instability in air or under the light, as well as their decreased solubility seriously thwart the development of novel higher acenes.^[^ ^5^ ^]^ In order to solve these problems, several strategies have been proposed and utilized. 1) Introducing electron‐withdrawing or bulky substituted groups such as silylethynyl, fluorine or phenylthio, at the active points on the backbones of acenes to enhance their stability.^[^ ^6^ ^]^ 2) Enabling the backbones of acenes twisted or π‐extended through the introduction of extreme steric shielding or benzannulated units (e.g., phenyl, pyrene, and triphenylene) to obtain the modified acenes (so‐called twistacenes or π‐extended acene derivatives), which are not fully consistent with the definition of acene.^[^ ^7^ ^]^ 3) In situ producing higher acenes in polymer matrix under the help of special UV light or on Au surface through heat treatment under ultrahigh vacuum.^[^ ^8^ ^]^ All the above‐mentioned strategies have already been demonstrated feasible to construct novel higher acenes. Aside from changing the substituted groups on the periphery of the conjugated backbone, another quite different strategy is to incorporate nonbenzenoid rings into acenes while maintaining their comparable conjugation length to form other types of high PCHs. Generally, these compounds could be realized via the replacement of the six‐membered benzenoid rings with other nonbenzenoid rings such as four‐membered, five‐membered, or even seven‐membered and eight‐membered ones.^[^ ^1^, ^9^ ^]^

**Figure 1:**
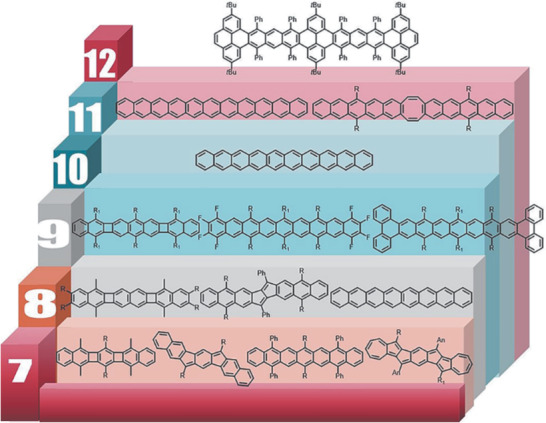
Some of polycyclic conjugated hydrocarbons with well‐defined structures discussed in this review (PCHs, *n* > 6).

Therefore, in this review, the state of the art to approach PCHs through the above‐mentioned four strategies are summarized (**Figure** **1**). Their synthetic methods are discussed first, followed by elaborating their basic physicochemical properties including photoelectronic properties, antiaromaticity and biradicals. In the final part, their single‐crystal packings and OFET performance will be briefly introduced, in consideration that the molecular packing modes usually have intimate relationship with the device performance. There are several points that are worth to be mentioned here. 1) This review will only focus on these PCHs only containing hydrogen and carbon elements on their main conjugated backbones, which means that many interesting PCHs containing heteroatoms (e.g., boron, nitrogen, oxygen or sulfur atom, etc.) will not be included in this review.^[^ ^10^ ^]^ 2) This review will mainly emphasize the linear‐fused PCHs, which are reported recently. Thus, the PCHs with 2D or graphene‐fragment‐liked structures will be excluded here.^[^ ^11^ ^]^ 3) This summary will only present the higher PCHs (*n* > 6) since the smaller PCHs (*n* < 7) have been elaborated in previous several reviews.^[^ ^1^, ^12^ ^]^

## 2 Synthetic Routes to PCHs

**Scheme 1:**
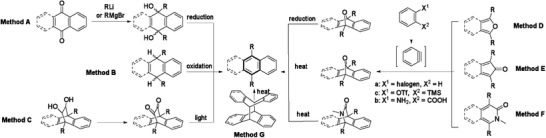
Brief summary of the synthetic methods to the linear PCHs containing benzenoid.

As shown in **Scheme** **1**, various methods to approach the linearly fused PCHs containing benzenoid have been summarized. In method A, the acenequinones are commonly used as starting materials for the preparation of acene derivatives through reacting with the nucleophilic organometallic reagents such as alkynyl lithium or Grignard reagent, followed by the reduction of the corresponding dihydroxylated moiety. This method is also frequently adopted to prepare the PCHs containing five‐membered or seven‐membered rings. In method B, the key intermediate hydroacenes are prepared first and then oxidized to the corresponding acenes. For methods C, D, E, and F, all key precursors contain a bridge as a leaving functional group (e.g., diketone, monoketone, oxygen atom or lactam group). In method C, the diketone bridge is transformed from dihydroxylated moiety and can be removed in polymer matrix under the irradiation of UV light. For methods D, E, and F, a general and effective way to construct the important precursors is to employ Diels–Alder reactions between the in situ formed benzyne (or bisbenzyne) and the appropriate dienes (furan, cyclopentadienone, isoquinolinone, and their derivatives). The corresponding acenes can be realized through the reduction or thermal elimination of these leaving groups. The methods to generate the in situ benzyne (or bisbenzyne) are convenient and can be accomplished by adopting different starting materials (e.g., halogen substituted compound for method D, *o*‐(trimethylsilyl)aryl triflates for method E and *o*‐aminoarenecarboxylic acid for method F). Method G presents a formal retro‐[4 + 4] cycloadditions utilizing the dimer precursor to prepare longer acenes.

The detailed PCH molecules discussed in Sections 2.1–2.4.

### 2.1 Substituted Acenes or π‐Extended Acene Derivatives

**Figure 2:**
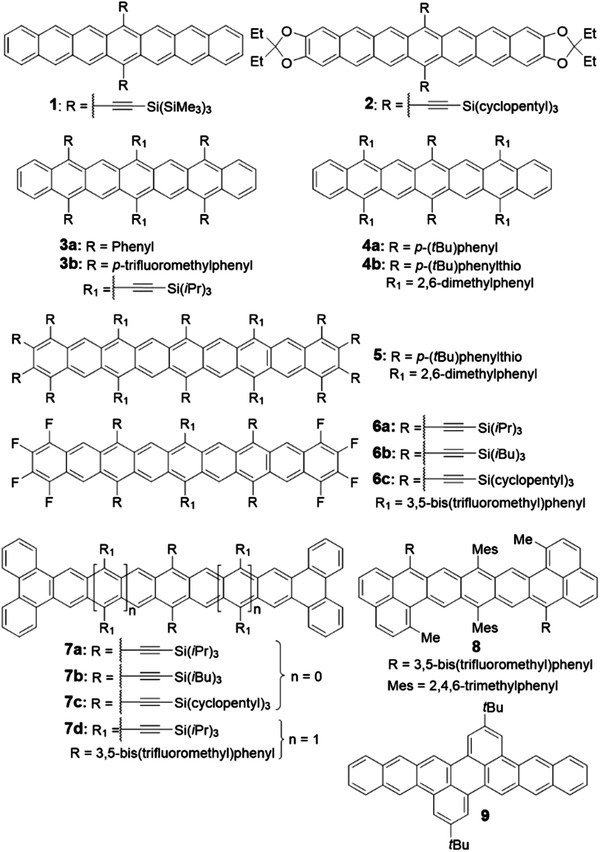
Structures of the substituted acenes or π‐extended acene derivatives.

**Scheme 2:**
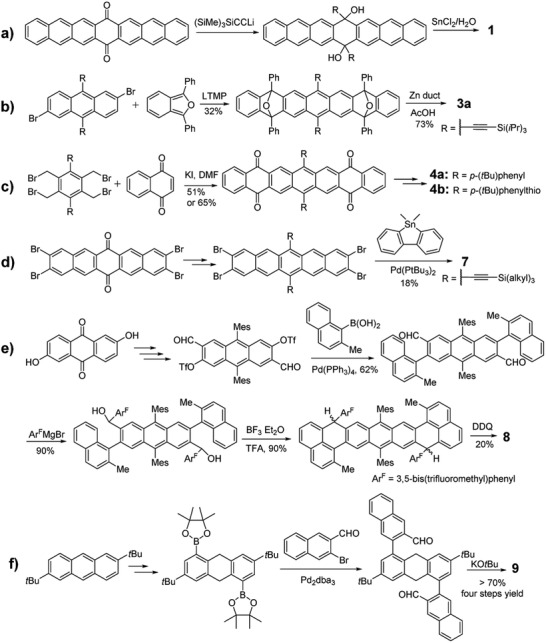
Synthetic routes to substituted acenes or π‐extended acene derivatives.

Poor solubility and instability are two main issues for the preparation and characterization of longer acenes (*n* > 6) as shown in **Figure** **2**. The specific decomposition reaction can be attributed to the oxidation by O_2_ to form endoperoxide products or the dimerization process under formal [4 + 4] cycloadditions.^[^ ^12b^ ^]^ To address them, one effective and widely used strategy is to introduce bulky‐protecting groups (trialkylsilylethynyl) at the periphery of the acene backbone. The three‐alkyl species on the silicon atom increase the solubility of acenes significantly. In addtion, the electron‐withdrawing silylethynyl group can not only retard oxidation, but also enable the endoperoxide formation reversible, thus dramatically enhancing their stabilities.^[^ ^6c^ ^]^ As shown in **Scheme** **2**a, Anthony group first used heptacenequinone as the starting agent to react with silylethynyl lithium, followed by the reduction with SnCl_2_ to produce the first stable functionalized heptacene **1** in 2005 (method A).^[^ ^5^ ^]^ Although its solution decomposed quickly when exposing in air, its single crystal remained stable for almost a week in laboratory conditions. Later, the same group synthesized dioxolane‐functionalized heptacene derivative **2**.^[^ ^13^ ^]^ However, this compound is too reactive to be separated and only its UV–vis–NIR absorption was measured. In 2008, as shown in Scheme 2b, a very stable substituted heptacene **3a** was synthesized in Wudl group through the reduction of the O‐bridged heptacene precursor, which was initially obtained by the reaction between 2,6‐dibromoanthracene and diphenylisobenzofuran in the presence of lithium reagent at low temperature (method D).^[^ ^14^ ^]^ Chi group synthesized a more stable substituted heptacene **3b** by introducing electron‐deficient 4‐trifluoromethylphenyl instead of phenyl groups.^[^ ^15^ ^]^ Later in 2009, as shown in Scheme 2c, Miller group employed 1,2,4,5‐tetrakis(bromomethyl)benzene and 1,4‐naphthoquinone as the precursors to construct the heptacene backbone containing four ketone groups via Diels–Alder reactions, which can be transformed into two novel functionalized heptacenes **4a** and **4b** by taking different nucleophilic phenyl or phenylthio lithium reactant.^[^ ^16^ ^]^ Compared to **4a** in solution, **4b** shows apparently better stability, indicating that apart from alkylsilylethynyl groups, other functional groups such as phenylthio‐substituents are also the appropriate blocks to stabilize higher acenes. Further study also indicates that the thioaryl substituents can significantly enhance the photooxidative resistance by rendering type‐II photooxidations less viable.^[^ ^16^, ^17^ ^]^ With the success in synthesizing **4b** containing the phenylthio‐substituent, the same group adopted the similar strategy to construct the ketone backbone of nonacene and then obtained the multiple phenylthio‐functionalized nonacene **5**.^[^ ^6a^ ^]^ However, the UV–vis–NIR and fluorescence spectra may indicate the formation of the decomposed byproduct of **5**. By replacing phenylthio groups with much electron‐withdrawing fluorine atoms and 3,5‐di(trifluoromethyl)phenyls and combining with bulky trialkylsilylethynyl groups, stable and fully characterized nonacene derivative **6a–c** were synthesized by Anthony group.^[^ ^6b^ ^]^ As shown in Scheme 2d, Bunz group also developed another strategy via stille coupling reaction to approach heptacene derivatives.^[^ ^18^ ^]^ The tetrabromopentacene was first synthesized by following the same synthetic route to introduce trialkylsilylethynyl group into compound **1** and then reacted with the dimethyl‐9‐stannafluorene to form the target molecules **7a–c**. Due to the four additional Clar sextets, **7a–c** are much stable under ambient conditions and only exhibit slight degradation after 2 h under UV lamp irradiation. In 2019, the same group prepared tetrabenzononacene **7d** by following the same synthetic method to prepare **4** and **5**.^[^ ^7c^ ^]^ Wu group also synthesized a series of dibenzo‐acene analogues.^[^ ^19^ ^]^ As shown in Scheme 2e, the key intermediate diformyl‐functionalized anthracene can be synthesized through multiple‐steps conversion from the starting material 2,6‐dihydroxyanthracene‐9,10‐dione, and can subsequently be subjected to react with (2‐methylnaphthalen‐1‐yl) boronic acid and Grignard reagent to afford the dihydroxylated precursor. Nonazethrene **8** was eventually obtained via an intramolecular Friedel–Crafts alkylation, followed by oxidative dehydrogenation approach.^[^ ^19c^ ^]^ Two groups prepared **9** with different synthetic method.^[^ ^20^ ^]^ As shown in Scheme 2f, Mastalerz group synthesized the key diborylated intermediated from 2,7‐di‐*tert*‐butylanthracene in two steps and reacted it with bromonaphthaldehyde to afford the dione precursor, which could be transformed into the target molecule **9** through the twofold condensation via treatment with KO*^t^*Bu.^[^ ^20a^ ^]^ Recently, Amsharov group also synthesized **9** through a modular approach by Pd‐catalyzed reaction between dihalogenarenes and formylareneboronic acids, followed by implementing a dehydrative π‐extension (DPEX) reaction.^[^ ^20b^ ^]^

**Figure 3:**
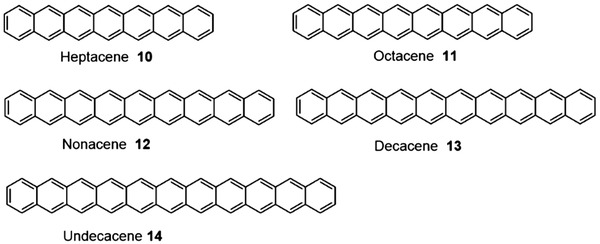
Structures of the unsubstituted acenes.

As far as we know, the substituted acenes higher than nonacene have not been reported probably due to the extreme instability and difficult synthesis. Meanwhile, through the above discussion, it is very clear that the linearly fused unsubstituted higher acenes will be even burdensome to obtain comparing with the linearly fused substituted higher ones. Through researchers’ consistent effect, longer acenes from heptacene **10** to undecacene **14** now can be observed in some matrix and on the surface of metal in ultrahigh vacuum as shown in **Figure** **3**.

### 2.2 Unsubstituted Acenes

**Scheme 3:**
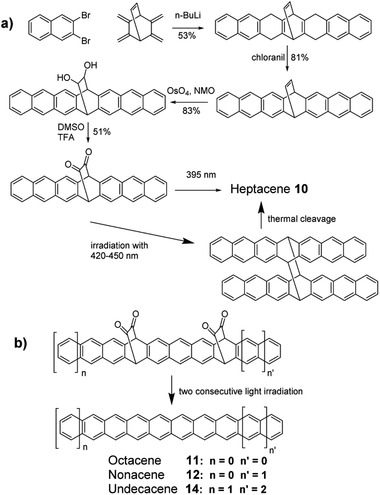
Synthetic routes to unsubstituted acenes through photogeneration.

As shown in **Scheme** **3**a and method C, the heptacene backbone with ethylene bridge was constructed through the reaction between the in situ formed active alkyne from 2,3‐dibromonaphthalene in the presence of *n*‐butyl lithium and the diene precursor bicyclo[2,2,2]oct‐2,3,5,6,7‐pentaene, then oxidizing via chloranil.^[^ ^8a^ ^]^ Afterward, the ethylene bridge was transformed into dihydroxyl group and diketone groups consequently. After burying in the PMMA film, heptacene **10** formed under a particular light irradiation and was confirmed by detecting its characteristic absorption in the UV–vis spectroscopy. Following the similar strategy, octacene **11**,^[^ ^21^ ^]^ nonacene **12**,^[^ ^21^ ^]^ and even undecacene **14** ^[^ ^22^ ^]^ were also photogenerated successfully. Compared with the formation of heptacene **10** irradiated with only UV light of 395 nm, the generation of all other three compounds **11**, **12**, and **14** needs irradiation under two different consecutive wavelengths of UV light in order to remove two groups of diketones step by step (Scheme 3b). In 2017, Bettinger group obtained a diheptacene through irradiating heptacenequinone, which further underwent thermal cleavage to form heptacene **10** confirmed by solid state NMR through a retro‐[4 + 4] cycloadditions process as depicted in Scheme 3a and method G.^[^ ^23^ ^]^

**Scheme 4:**
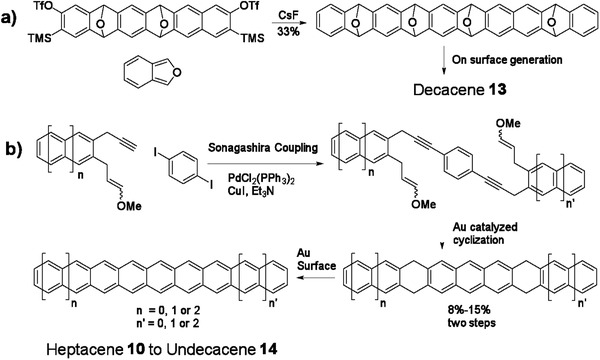
Synthetic routes to unsubstituted acenes through on surface generation.

**Figure 4:**
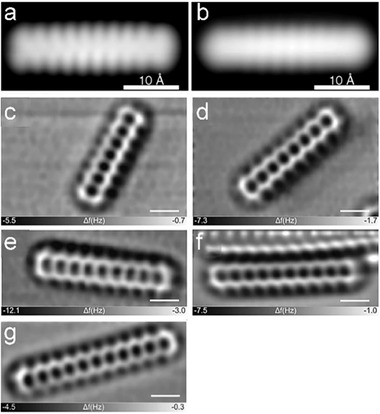
a,b) Constant‐current STM images of **13** acquired at a sample voltage of *V* = −0.4 V (a), and −0.8 V (b), respectively. c–g) Frequency shift nc‐AFM images of **10–14** generated by annealing. Reproduced with permission.^[^ ^8^, ^22^ ^]^ Copyright 2017 and 2018, John Wiley & Son.

Aside from the photogeneration of higher acenes from diketone precursors, these compounds can also be prepared on Au surface through the reduction of O‐bridged precursor (method D).^[^ ^24^ ^]^ As shown in **Scheme** **4**a, the tetraepoxy decacene precursor was synthesized via multiple steps of [4 + 2] cycyloaddition reaction between isobenzofuran and the in situ formed alkyne derived from the precursor containing neighboring —OTf and —TMS groups. Afterward, it was sublimated onto the Au surface and converted into decacene **13**, whose constant‐current STM images were recorded after tip‐assisted synthesis as shown in **Figure** **4**a,b.^[^ ^8b^ ^]^ In 2018, Echavarren group adopted an interesting Au‐catalyzed cyclization reaction to construct the hydroacene with various lengths from linearly fused 7 to 11 rings as drawn in Scheme 4b.^[^ ^25^ ^]^ They can be further oxidized into various acenes from heptacene **10** to undecacene **14** according to method B.^[^ ^26^ ^]^ Since simple STM imaging was not sufficient to further discern the details of the adsorbates in atomic level, high‐resolution noncontact atomic force microscopy (nc‐AFM) was utilized to testify undoubtedly the formation of this serial of acenes from **10** to **14** (Figure 4c–g). Gottfried group also prepared heptacene **10** on Ag(111) surface from a method by surface‐assisted didecarbonylation of the α‐diketone precursor.^[^ ^27a^ ^]^ Recently, on‐surface formation of heptacene **10** and nonacene **12** was also realized via visible‐light‐induced photodissociation of α‐bisdiketone precursors on Au(111) substrate under ultrahigh vacuum conditions.^[^ ^27b^ ^]^

### 2.3 Pyrene‐Fused π‐Extended Acene Derivatives

**Figure 5:**
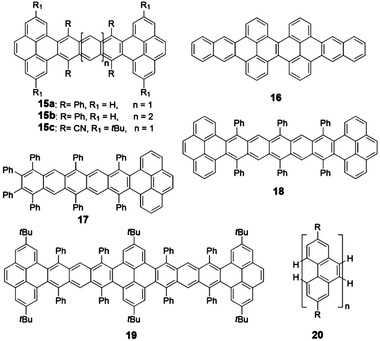
Structures of pyrene‐fused π‐extended acene derivatives.

Apart from the above‐mentioned synthetic strategies, researchers are also exploiting other building blocks to acquire stable acenes. Due to its outstanding photoelectrochemical properties and conjugation character, pyrene has been widely used as a building unit to construct various organic electronic materials.^[^ ^28^ ^]^ In addition, pyrene is also employed as the building block to synthesize PCHs.^[^ ^29^ ^]^ As shown in **Figure** **5**, various stable and twisted linearly fused higher acenes or arenes containing pyrene units were synthesized by attaching multiple phenyls as the functional groups.

**Scheme 5:**
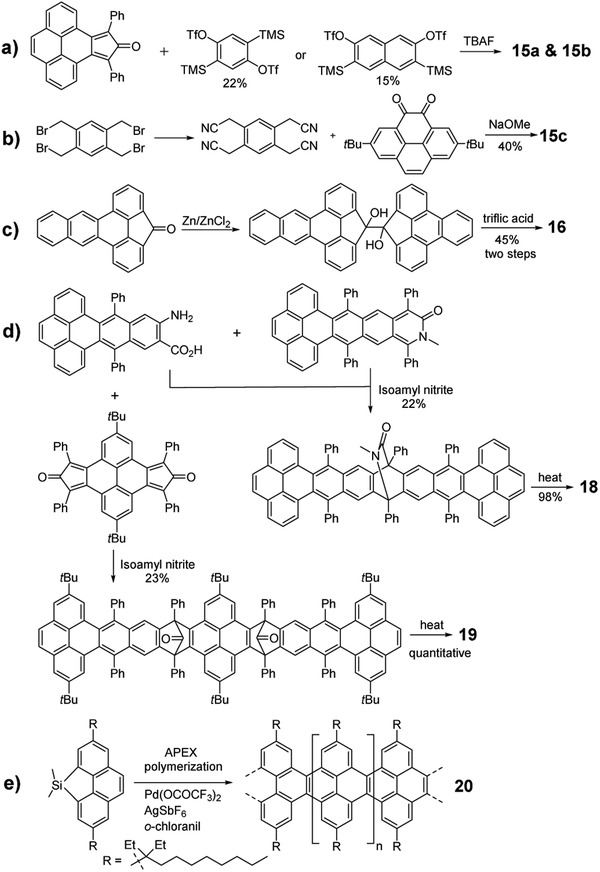
Synthetic routes to pyrene‐fused π‐extended acene derivatives.

As shown in **Scheme** **5**a, Wudl group used 2,5‐bis(trimethylsilyl)‐1,4‐phenylene bis(trifluoromethanesulfonate) as the bisbenzyne precursor and pyreno‐diphenylcyclopentadienone as the diene to successfully synthesize heptatwistacene **15a** in one step in the presence of TBAF.^[^ ^30a^ ^]^ By adopting the same strategy, Zhang and Pena groups reported the synthesis of octatwistacene **15b** by changing the aryne precursor to 3,6‐bis(trimethylsilyl)naphthalene‐2,7‐diyl bis(trifluoromethanesulfonate).^[^ ^30b,c^ ^]^ Compared with **15a**, compound **15c** with four cyano functional groups was synthesized under the base condition between 2,7‐di‐*tert*‐butylpyrene‐4,5‐dione and 1,2,4,5‐tetrabromomethylbenzene, which was transformed from tetracyanomethylbenzene (Scheme 5b).^[^ ^30d^ ^]^ As shown in Scheme 5c, Tao group synthesized the tetrabenzoacene **16** in anhydrous benzene containing triflic acid from the pinacol precursor, which was prepared from the fluorenone derivative by pinacol coupling in the presence of Zn/ZnCl_2_.^[^ ^31^ ^]^ Zhang group also synthesized a series of twistacenes including **17** ^[^ ^32^ ^]^ and **18** ^[^ ^7b^ ^]^ by using a so‐called “clean reaction” as indicated in method F. As shown in Scheme 5d, this method used *o*‐aminoarenecarboxylic acid as the aryne precursor and isoquinolinone as the diene to obtain a lactam‐bridged precursor, which was further converted into the corresponding nonatwistacene **18** in almost quantitative yield by thermal elimination of the bridge in a sealed high‐vacuum tube. Recently, the same group synthesized a dodecatwistacene (**19**) using a similar way to method E.^[^ ^33^ ^]^ The key intermediate with two ketone bridges was prepared from the *o*‐aminoarenecarboxylic acid and dicyclopenta[e,l]pyrene‐5,11‐dione through a twofold [4 + 2] cycloaddition (Scheme 5d). Then, compound **19** was obtained through thermal treatment, which is the longest linearly fused twisted arene with single‐crystal structure reported so far. Aside from the linearly fused pyrene‐contained acenes or arenes, it is worth mentioning that Itami and co‐workers recently synthesized a fused polypyrene **20**, which incorporates pyrenes onto a linear arene scaffold to the extreme via the annulative π‐extension (APEX) strategy (Scheme 5e).^[^ ^34^ ^]^ The homo‐polymerization enables the synthesis of cove‐type graphene nanoribbons with precisely controlled widths and edge structures in a single operation.

**Figure 6:**
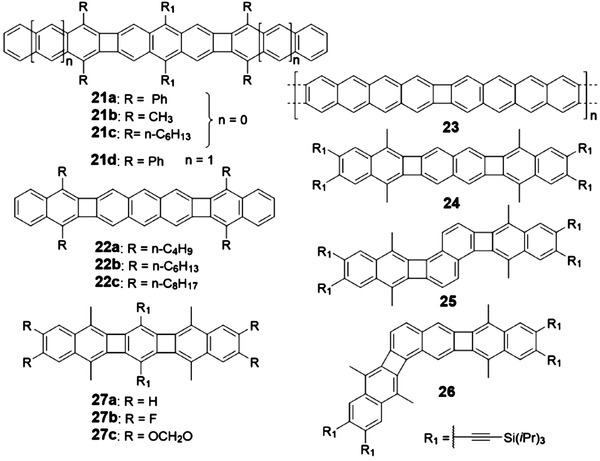
Structures of PCHs containing CBD.

**Figure 7:**
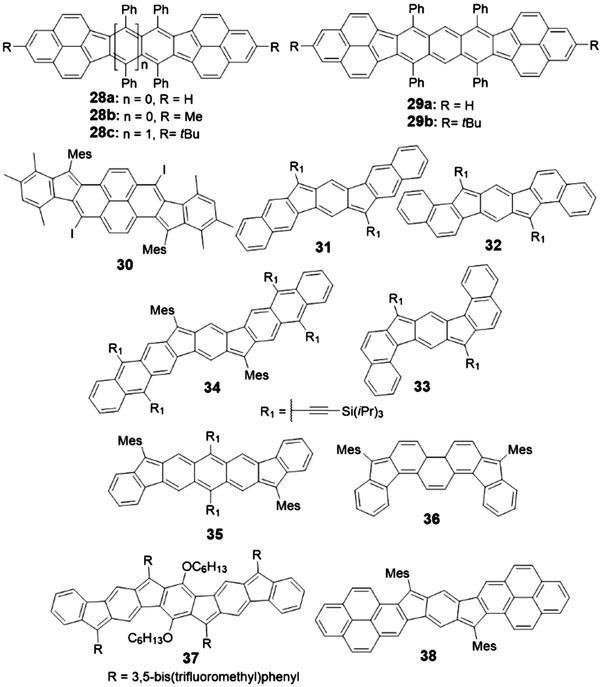
Structures of PCHs containing indacene.

**Figure 8:**
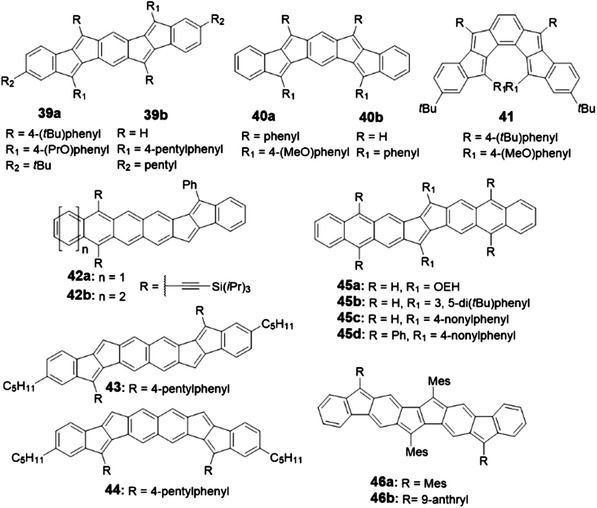
Structures of PCHs containing pentalene.

**Figure 9:**
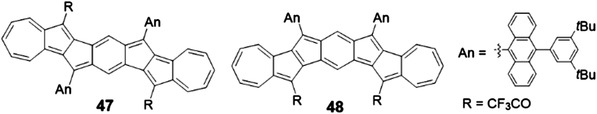
Structures of PCHs containing azulene.

**Figure 10:**
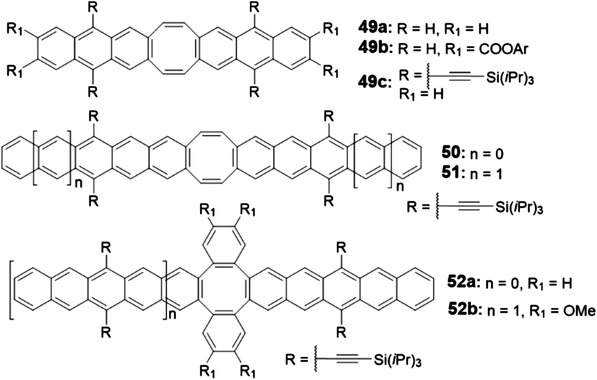
Structures of PCHs containing COT.

The above discussion mainly focuses on PCHs that consist of acenes with fused benzenoid rings. Although these molecules have drawn great attention in the early stage for developing organic semiconductors, their inherent sensitivity to ambient condition to form the photooxidization or dimerization product motivates researchers to develop the acene alternatives that can not only maintain the promising electronic properties of acenes but also possess much improved stability. One effective strategy is to replace the benzenoid ring with other nonbenzenoid membered ring. As shown in **Figures** **6**–**10**, the state of the art PCHs or oligoacenes containing four‐membered, five‐membered, seven‐membered, or even eight‐membered rings are synthesized recently.

### 2.4 Linearly Annulated PCHs with Nonbenzenoid Rings

#### 2.4.1 PCHs with Four‐Membered Ring‐Cyclobutadienoid (CBD)

**Scheme 6:**
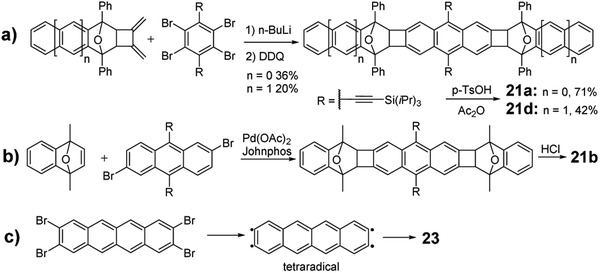
Synthetic routes to PCHs containing CBD.

Vollhardt group developed the cobalt‐catalyzed [2 + 2 + 2] cycloaddition to approach various six‐membered benzenoid‐contained molecules with linear, zigzag, or helical shapes, where the four‐membered CBD as the linker was simultaneously formed.^[^ ^35^ ^]^ As shown in **Scheme** **6**a, in 2012, Swager group reported the synthesis of linear PCH molecules **21a** and **21d** with CBD rings.^[^ ^9a^ ^]^ The in situ formed bis‐functionalized dienophile/diaryne reacted with the diene, synthesized from 1,3‐diphenylisobenzofuran or 1,3‐diphenylisonaphthofuran, to produce the O‐bridged intermediates, which could be transformed into the linear oligoacene molecules **21a** and **21d** in the acid condition. Later, as shown in Scheme 6b, Xia group developed a novel Pd‐catalyzed reaction utilizing Johnphos phosphorus ligand between 1,4‐dimethyl‐1,4‐dihydro‐1,4‐epoxynaphthalene and functionalized 2,6‐dibromoanthracene to construct the key intermediate, which can be aromatized into **21b** via a dehydration reaction in the condition of strong HCl acid.^[^ ^36^ ^]^ By employing the same strategy, Xia group synthesized another two series of molecules **24–26** ^[^ ^37a^ ^]^ and **27a–c** ^[^ ^37b^ ^]^ by changing the core units from anthracene to naphthalene and benzene. Similarly, Miao group also synthesized the similar molecules **22a–c** with different alkyl groups and investigated their influence on the hole mobility.^[^ ^38^ ^]^ Recently, Fasel group utilized the on‐surface synthesis strategy to afford an acene‐based nanoribbons incorporating CBD rings (Scheme 6c). 2,3,8,9‐Tetrabromotetracene is activated on the Ag(111) surface through a dehalogenation reaction to form the tetraradical, which subsequently undergoes a formal [2 + 2] cycloaddition reaction to produce **23** at 475 K.^[^ ^39^ ^]^

PCHs with five‐membered rings have been investigated intensively. They mainly contain indacene (Figure 7)^[^ ^1^, ^12^, ^40^ ^]^ or pentalene motifs (Figure 8).^[^ ^40^, ^41^ ^]^ For indacene‐based molecules, especially for indenofluorenes, Haley group has discussed the synthetic strategies in detail, dubbed “inside‐out” or “outside‐in” according to the condition whether the C=O unit (including acid, ester or aldehyde) is appended onto the central ring or outer ring first.^[^ ^1f^ ^]^ For pentalene‐based molecules, various synthetic methods including Ni‐, Pd‐ or Au‐catalyzed annulation,^[^ ^42^ ^]^ Lewis acid‐induced intramolecular reaction^[^ ^43^ ^]^ and other anionic or radical anionic‐involved annulation^[^ ^44^ ^]^ have been reported and summarized in several reviews.^[^ ^41b,c,h^ ^]^ Therefore, taking into consideration of the necessity and the convenience to compare with other higher PCHs with four‐, six‐, seven‐ or eight‐membered ring in the following sections, in this part, we will only focus on higher PCHs (*n* > 6) with five‐membered ring and highlighting their corresponding synthetic routes, although many novel and interesting bispentalene and dibenzopentalene compounds in shorter length (*n* < 6) have been published recently.^[^ ^41^, ^45^ ^]^

#### 2.4.2 PCHs with Five‐Membered Ring‐Indacene

**Scheme 7:**
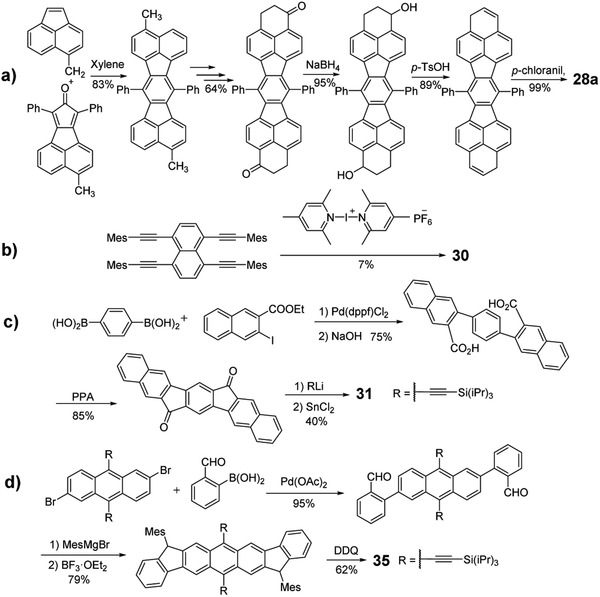
Synthetic routes to PCHs containing indacene.

For indacene‐based molecules, as shown in **Scheme** **7**a, to obtain phenalenyl‐based **28a**, the key backbone was first constructed through Diels–Alder reaction and the —CH_3_ group was converted into —CH_2_Br, —CH_2_CH_2_COO*^t^*Bu, —CH_2_CH_2_COOH, and —CH_2_CH_2_COCl consecutively. Intramolecular Friedel–Crafts cyclization of the acyl chloride with AlCl_3_ produced the cyclic diketone compound, which was reduced, dehydrated and subsequently oxidized by *p*‐chloranil to afford **28a**.^[^ ^46^ ^]^ By changing the group —CH_2_CH_2_COO*^t^*Bu to —CH_2_CH(CH_3_)COO*^t^*Bu, **28b** was obtained.^[^ ^47^ ^]^ Naphthalene‐ and anthracene‐linked compounds **28c**, **29a**, and **29b** were synthesized following the similar idea to prepare **28a**.^[^ ^48^ ^]^ Interestingly, the reaction between 1,4,5,8‐tetrakis(mesitylethnyl)naphthalene and bis(2,4,6‐trimethylpyridine)iodine(I) hexafluorophosphate yielded compound **30** (Scheme 7b).^[^ ^49^ ^]^

As shown in Scheme 7c, compound **31** was obtained through nucleophile addition of the corresponding lithium salt to the dione precursor, followed by the reductive dearomatization of the intermediate diol.^[^ ^50^ ^]^ The dione intermediate can be synthesized through three steps reactions: 1) coupling reaction between the boronic acid and halogenated naphthalene; 2) saponification reaction; and 3) ring closure reaction catalyzed by polyphosphoric acid. The isomers **32** and **33** were synthesized according to the same way by using the different‐position halogenated naphthalene.^[^ ^50^ ^]^ In 2016, as shown in Scheme 7d, Haley group used functionalized 2,6‐dibromoanthracene and 2‐formylbenzeneboronic acid as starting materials to form the key aldehyde intermediates, which were transformed into the target molecule **35** through the nucleophilic reaction, Friedel–Crafts alkylation mediated by boron trifluoride etherate, and oxidization reaction.^[^ ^51^ ^]^ It is worth to mention that the overall yield for these four steps reaction is as high as 49%. Compounds **34** and **36–38** can also be obtained according to the similar synthetic strategy for **35** by just changing the starting materials into different bromo‐substituted arylaldehydes and different arylboronic acids or ester.^[^ ^52^ ^]^

#### 2.4.3 PCHs with Five‐Membered Ring‐Pentalene

**Scheme 8:**
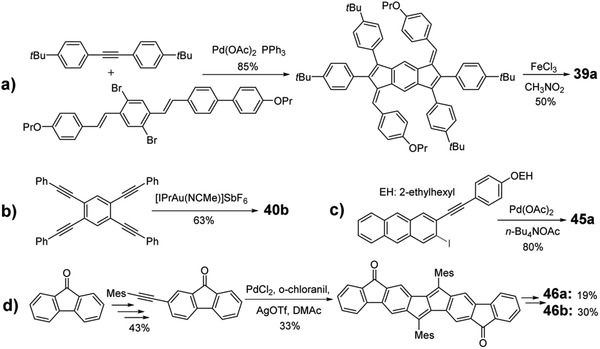
Synthetic routes to PCHs containing pentalene.

In 2010, by taking the classic Heck reaction, Tilley group reacted the bromostilbene derivatives with excessive diarylacetylene to afford the key indacene intermediate, which could be transformed into linear dipentalene **39a** by using FeCl_3_ as the oxidative reagent as shown in **Scheme** **8**a. By changing different bromostilbene derivatives, semilinear dipentalene **40a** and bent dipentalene **41** were also obtained.^[^ ^53^ ^]^ Dipentalene compound **40b** was also successfully synthesized by taking the tetraalkyne‐contained precursor (Scheme 8b).^[^ ^42f^ ^]^ Acenopentalenes **42a** and **42b** can be obtained by using the same catalysis.^[^ ^54^ ^]^ Later, the same group found out that the shape of the dipentalene isomers could be tuned through adjusting the bulkiness of the ligand and the substrates. In addition to **39b** and **40b**, naphthalene‐linked dipentalenes **43** and **44** were also selectively approached.^[^ ^55^ ^]^ Pd‐catalyzed reaction is another prevalent method to construct the pentalene unit‐based functional molecules.^[^ ^42^, ^56^ ^]^ As shown in Scheme 8c, Zhu group developed a heterogeneous catalytic system, Pd(OAc)_2_/*n*‐Bu_4_NOAc, to effectively produce diaceno[a,e]pentalenes **45a** via a tandem Pd catalytic cycle by using *o*‑alkynylaryliodide as a substrate.^[^ ^56a^ ^]^ Later, the same group shortened the synthesis route to one step by directly reacting 2,3‐dibromoanthracene with tributyl(arylethynyl) stannanes.^[^ ^56b^ ^]^ By changing different substrates, the dibenzo[a,e]pentalene (DBP), dinaphtho[a,e]pentalene (DNP), and dianthraceno[a,e]pentalene (DAP, **45b**) were constructed, and their corresponding OFET device performances were systematically evaluated.^[^ ^57^ ^]^ Chi group took 2‐bromo‐3‐((4‐nonylphenyl)ethynyl)anthracene as the reactant and Pd_2_(dba)_3_ as the catalysis to yield the dimerized products **45c** and **45d**.^[^ ^58^ ^]^ Itami group synthesized **46** in six steps starting from the fluorenone as drawn in Scheme 8d. The intermediate 2‐(mesitylethynyl) fluorenone could be prepared through bromination and subsequent Sonogashira cross‐coupling was subjected to Pd‐catalyzed annulation to form the pentalene backbone with dione units. After further introducing the regarding mesityl or 9‐anthryl group into the dione positions and the reduction reaction, **46** was successfully obtained.^[^ ^42e^ ^]^

#### 2.4.4 PCHs with Seven‐Membered Ring‐Azulene

**Scheme 9:**
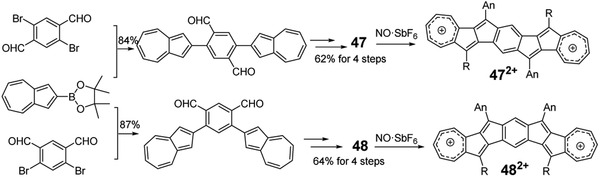
Synthetic routes for PCHs containing azulene.

Aside from the four‐membered‐ and five‐member‐ring fused PCH molecules, seven‐membered‐ring fused PCHs also achieved significant development recently.^[^ ^59^ ^]^ As shown in Figure 9 and **Scheme** **9**, by adopting the similar synthetic route to **35**, Chi group synthesized two novel seven‐membered‐ring fused molecules **47** and **48** by taking azulen‐2‐ylboronic acid pinacol ester as the starting agents.^[^ ^60^ ^]^ Interestingly, these molecules can change to their dication forms after the treatment with NO·SbF_6_ reagent.

#### 2.4.5 PCHs with Eight‐Membered Ring‐Cyclooctatetraene (COT)

**Scheme 10:**
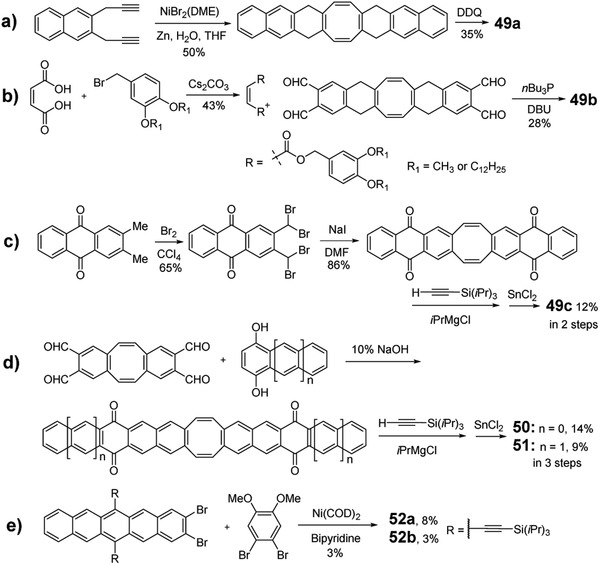
Synthetic routes for PCHs containing COT.

PCHs containing 8π COT ring have been studied recently by Saito group.^[^ ^61^ ^]^ The molecules show environmentally dependent RGB luminescence and can be utilized as photofunctional materials such as molecular viscosity probes. However, these molecules contain the imide groups in terminal rings, which may significantly change their basic properties compared with other PCHs containing only C and H elements in the backbone as shown in Figure 10. Hence, these molecules will not be further elaborated in this review. Later, as shown in **Scheme** **10**a, unsubstituted **49a** was synthesized through the metal‐mediated [2 + 2 + 2 + 2] cycloaddition^[^ ^62^ ^]^ of a terminal naphthalene‐based diyne, followed by DDQ oxidation.^[^ ^63^ ^]^ The tetraester‐functionalized **49b** was synthesized by taking the acene‐elongation strategy through reacting an olefin with an aromatic *o*‐dialdehydes (Scheme 10b).^[^ ^63a^ ^]^ The trialkylsilylethynyl‐substituted compounds **49c**, **50**, and **51** were transformed from the critical ketone intermediate, which was derived from 2,3‐bis(dibromomethyl)anthraquinone^[^ ^64^ ^]^ and dibenzo[a,e]COT‐based tetraaldehyde,^[^ ^63b^ ^]^ respectively (Scheme 10c,d). Recently, as shown in Scheme 10e, Bunz group used the Yamamoto coupling to obtain an acene‐based cyclooctatetraenes **52a** and **52b** by the combination of dibromoacenes and dibromobenzenes, although their yields were 8% and 3%, respectively.^[^ ^65^ ^]^

## 3 Physicochemical Properties and Single Crystal Structures of Some Above‐Mentioned PCHs

### 3.1 Optoelectrical Properties

**Figure 11:**
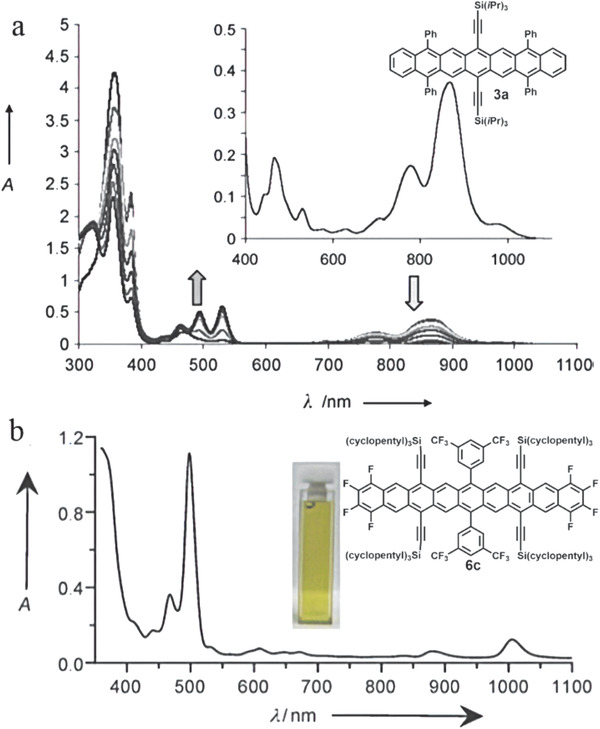
a) UV–vis–NIR degradation study of functionalized heptacene **3a** in toluene under ambient conditions. An inset is the absorption in 0 min. b) UV–vis–NIR absorption of nonacene **6c**. Reproduced with permission.^[^ ^6^, ^14^ ^]^ Copyright 2011 and 2008, John Wiley & Sons.

Heptacene **1** has the *λ*_onset_ absorption of 912 nm and a calculated optical bandgap of 1.36 eV, which corresponds well to its electrochemical bandgap of 1.30 eV (the oxidation peak: 0.470 V versus SCE and reduction peak: −0.830 V versus SCE).^[^ ^5^ ^]^ Different solvents affect the molecular aggregation formation and *λ*_max_ blue‐shifts from 852 nm in polar solvent CH_2_Cl_2_ to 835 nm in nonpolar solvent Hexane. Similar to molecule **1**, although compound **3a** possesses additional four phenyl groups, it still has a typical heptacene *λ*_onset_ at 917 nm (equals to an optical bandgap of 1.35 eV), which is also similar to the value measured through cyclic voltammetry (1.38 eV) (**Figure** **11**a).^[^ ^14^ ^]^ The continuously increased absorption intensities at 535 nm and UV region suggest the formation of a series of dioxygen‐contained adducts. Compound **4a** decomposed very quickly in solution while **4b** showed much improved stability. Its longest‐wavelength absorption *λ*_max_ is 865 nm and the *λ*_onset_ absorption is 905 nm, suggesting an optical bandgap of 1.37 eV.^[^ ^16^ ^]^ Compound **5** with the protection of multiple phenylthio groups exhibited a much smaller optical bandgap of 1.12 eV.^[^ ^6a^ ^]^ Through careful selection of the substituted groups such as trifluoromethylphenyl and silylethynyl as well as fluorine atoms, nonacenes **6a–c** were successfully synthesized.^[^ ^6b^ ^]^ As shown in Figure 11b, these compounds have the absorption reaching to 1033 nm and the optical bandgap of 1.20 eV. Compounds **7a–c** display *λ*_max_ at about 760 nm, which is larger than that of hexacene (*λ*_max_ = 738 nm) but lower than that of heptacene (*λ*_max_ = 835 nm), indicating that two triphenylene units contribute to the bathochromic shift of hexacene.^[^ ^5^, ^18^ ^]^ Acene‐elongated tetrabenzononacene **7d** exhibited similar optical absorption with *λ*_max_ of 958 nm, smaller than those of nonacenes **6a–c** (1014 nm).^[^ ^7c^ ^]^

**Figure 12:**
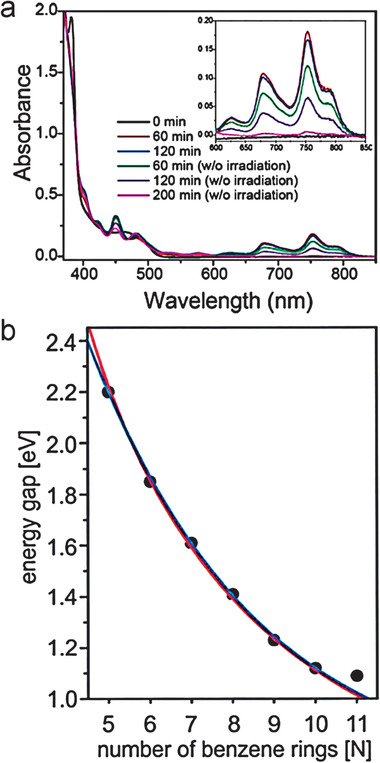
a) Absorption spectra of heptacene **10** after irradiating the diketone precursor in a PMMA film (inset: enlarged portion from 600 to 850 nm). b) Energy gap versus number of benzene rings measured through scanning tunneling spectroscopy (STS). Reproduced with permission.^[^ ^8^, ^26^ ^]^ Copyright 2006, American Chemical Society.

Irradiation of the PMMA film containing the diketone precursor with the UV‐LED array produced a new absorption extending from 600 to 825 nm with *λ*_max_ at 760 nm as shown in **Figure** **12**a, demonstrating the formation of heptacene **10** with an optical bandgap of 1.50 eV.^[^ ^8a^ ^]^ Its intensity increased significantly in the first 60 min but exhibited no additional enhancement with further irradiation. Meanwhile, the intensity decreased even without irradiation, indicating the inherent instability of **10**. For compounds **11**, **12**, and **14**, their characteristic *λ*_max_ red‐shifted to 806, 865, and 1007 nm, respectively, indicating a gradually reduced energy gap as the conjugated length increases.^[^ ^21^, ^22^ ^]^ Scanning tunneling spectroscopy (STS) measurements provide HOMO–LUMO gaps of 1.50 eV for heptacene **10**, 1.25 eV for nonacene **12**, and 1.17 eV for decacene **13** on Au(111).^[^ ^8^, ^27^ ^]^ Echavarren group used the STS technology to confirm the energy gaps of the series of acenes from **10** to **14** and summarized the detailed relationship between the HOMO/LUMO gaps and the number of benzene rings. As shown in Figure 12b, both the real inverse proportionality (red line) from pentacene to decacene and the exponential decay (blue line) could be well fitted, however, undecacene deviates toward a larger gap value, indicating the increased contribution of the open‐shell configuration and saturation of lowering bandgap for higher acenes.^[^ ^26a^ ^]^

For three pyrene‐fused twisted molecules **15a**,^[^ ^30a^ ^]^ **15b**,^[^ ^30b,c^ ^]^ and **18**,^[^ ^7b^ ^]^ they exhibited gradually red‐shifted *λ*_max_ from 530 to 618 and 739 nm, corresponding to the decreased energy gaps from 2.50 to 2.09 and 1.72 eV, respectively, as the number of conjugated benzyl rings increases. It is worth to mention that the bandgap of **17** (1.92 eV) derived from electrochemical measurement is even larger than the bandgap of a typical hexacene (1.84 eV), indicating that the twisted structure weakens the conjugation of the whole arene backbone.^[^ ^32^ ^]^ When changing the four phenyl groups into four more electron‐withdrawing cyano groups, **15c** showed much red‐shifted *λ*_max_ at 577 nm than that of **15a** (530 nm).^[^ ^30d^ ^]^ Due to the low solubility of **16** in common solvents, its UV–vis absorption spectra was not obtained.^[^ ^31^ ^]^

**Figure 13:**
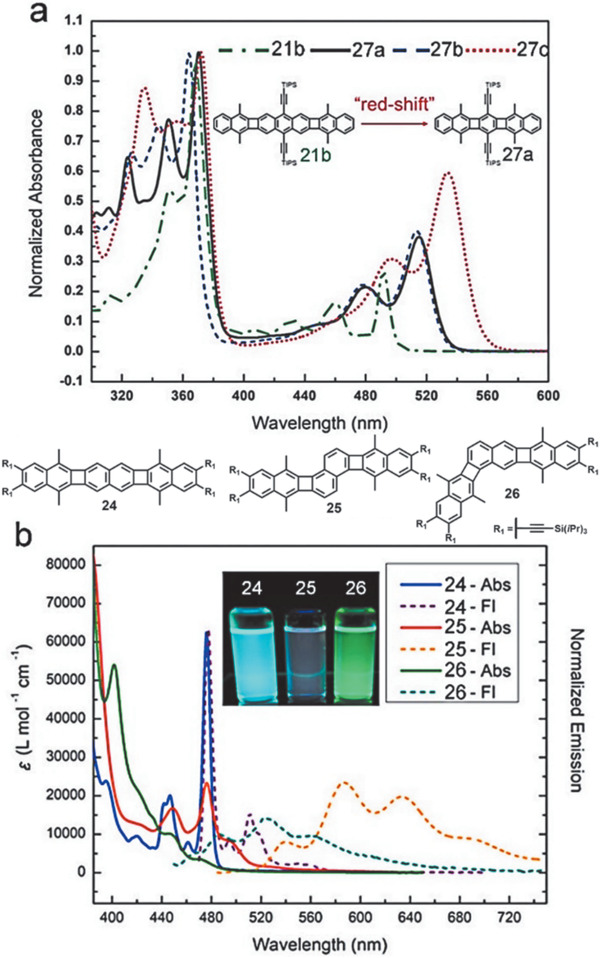
a) UV–vis spectra of **21b** and **27a–c** in CHCl_3_. b) UV–vis and fluorescence spectra of **24–26**. Reproduced with permission.^[^ ^37a,b^ ^]^ Copyright 2017, American Chemical Society and Copyright 2019, John Wiley & Sons.

As shown in **Figure** **13**a, compound **21b** can be considered as the central TIPS–anthracene connected with two naphthalene parts through two four‐membered rings.^[^ ^9^, ^36^, ^37^ ^]^ Compared with TIPS–anthracene (2.74 eV), the optical energy gap of **21b** decreased to 2.48 eV with a *λ*_max_ around 500 nm, about 0.26 eV smaller than that of TIPS–antharacene, suggesting that there is still sufficient interaction between two antharacene and naphathalene chromophors, despite that phenylene linkage restricts the electron delocalization through the whole molecule. Compound **21d** with extension of the outer acenes had bathochromic shift *λ*_max_ of 513 nm compared with **21a**.^[^ ^9a^ ^]^ Interestingly, compared with **21b**, compound **27a**, which contains only one benzenoid ring as the central core, counterintuitively exhibited a longer wavelength *λ*_max_ of 515 nm and *λ*_onset_ at 534 nm, corresponding to an optical bandgap of 2.32 eV, lower than that of **21b** at 2.48 eV.^[^ ^37b^ ^]^ The fluorescence of **27a** and **21b** displayed small Stokes shift of only 11 and 2 nm, but relatively high quantum yields of 64% and 75%, respectively. Similar to the UV–vis absorption of **21b** and **27a**, the same phenomenon also happened in **22b** and **24**. The optical bandgaps decrease slightly from 2.64 eV for **22b** to 2.56 eV for **24** when the central part changes from anthracene to naphthalene.^[^ ^37a^ ^]^ For compounds **21c** and **22b** which contain the *n*‐hexyl functional groups, due to the extended conjugation and withdrawing effect of ethynyl group in **21c**, **21c** exhibits a ≈27 nm red‐shifted longest‐wavelength *λ*_max_ of 493 nm compared with that of **22b**, corresponding to a lower optical bandgap of 2.48 eV, similar to that of **21a** (2.43 eV). Nevertheless, the tetra‐fluorination substituted compound **27b** displayed an almost identical profile to **27a** in the visible region at 515 nm while the more‐electron‐rich compound **27c** with dioxalane substituents red‐shifted to 534 nm (*λ*_max_).^[^ ^37b^ ^]^ Aside from the functional groups, the molecule shapes also affect the absorption significantly. As shown in Figure 13b, although the linear isomer **24** and bent isomer **26** exhibited a similar *λ*_onset_ (484 and 489 nm) and optical bandgaps (2.56 and 2.54 eV), the absorption intensity was much smaller for **26** compared with **24** in the 450–500 nm region. In addition, the angular isomer **25** exhibited much bathochromic shift of *λ*_onset_ (extending to 550 nm) than both of the other two isomers **24** and **26** and a much‐decreased optical bandgap of 2.25 eV.^[^ ^37a^ ^]^ These isomers also exhibited distinct emission profiles and quantum yields. As shown in Figure 13b, compounds **25** and **26** display apparently broad emission and large Stokes shift compared with **24**, which has a sharp emission with only 1 nm Stokes shift. In addition, as shown in the inset, **24** shows much stronger fluorescence emission than the other two isomers containing angular architecture. Actually, the quantum yield decreases sharply from 0.64 for **24** to 0.16 for **26** and only 0.08 for **25**.^[^ ^37a^ ^]^

**Figure 14:**
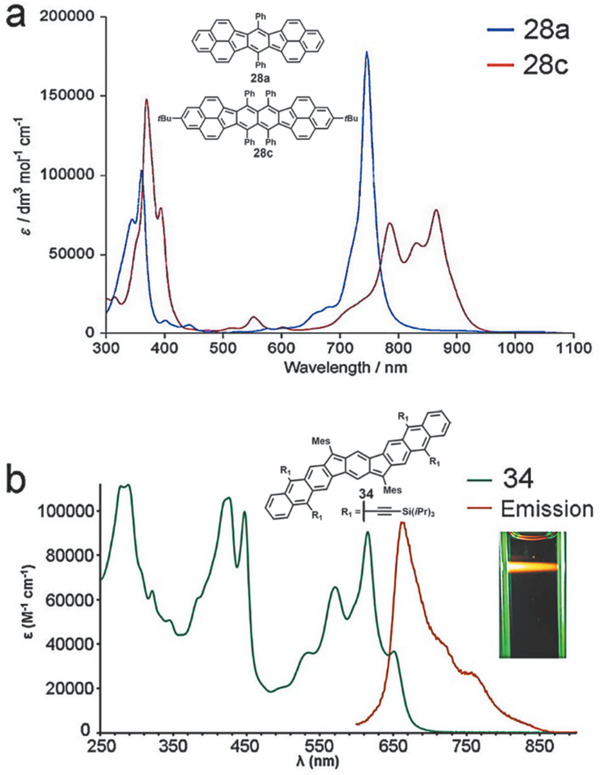
a) UV–vis spectra of **28a** and **28c**. b) UV–vis and fluorescence spectra of **34**. Reproduced with permission.^[^ ^48^, ^52^ ^]^ Copyright 2007, American Chemical Society and Copyright 2018, Thieme Medical Publishers.

As shown in **Figure** **14**a, for the indacene‐based PCHs containing five‐membered ring with terminal phenalenyl units, all three compounds **28a**, **28c**, and **29b** exhibit similar UV–vis absorption profiles containing two main absorption regions (300–450 and 700–1000 nm).^[^ ^47^, ^48^ ^]^ As their conjugation increases from **28b** to **28c** and **29b**, their lowest energy *λ*_max_ increases simultaneously from 746 to 865 and 984 nm. Meanwhile, their electrochemical bandgaps decrease gradually from 1.20 to 1.04 and 0.98 eV, corresponding well with the decreased optical bandgaps tendency from 1.64 to 1.43 and 1.26 eV, measured from the optical UV spectra. Compared with the *λ*_onset_ of **27a** containing four‐membered CBD ring in around 530 nm, the linear indenofluorene‐based **31** exhibits much red‐shifted *λ*_onset_ absorption (633 nm), demonstrating the improved delocalization for acene analogues containing five‐membered ring.^[^ ^50^ ^]^ The isomers **31**, **33**, and **32** displayed gradually red‐shifted *λ*_onset_ absorption from 633 to 648 and 667 nm, indicating that **32** has the smallest optical bandgap of 1.86 eV among these three isomers. In comparison with **31**, the acene‐extended **34** exhibits a red‐shifted absorption *λ*_max_ at 615 nm and a shoulder peak at 651 nm as shown in Figure 14b.^[^ ^52d^ ^]^ Nevertheless, the *λ*_onset_ value of **35** reaches to 900 nm in the near infrared region. The deep‐blue color of **35** in solution could be ascribed to an absorption at 690 nm that extends into green and red regions of the visible spectrum.^[^ ^51^ ^]^ Compared with **35**, **36** exhibits an even longer wavelength (*λ*_onset_ at 1050 nm), corresponding to an even lower energy gap of 1.18 eV.^[^ ^52a^ ^]^ The solution of **37** in DCM shows an intense *λ*_max_ at 722 nm with a long tail extending beyond 1000 nm.^[^ ^52c^ ^]^ Its electrochemical energy gap is estimated to be 1.37 eV, which corresponds well with its optical energy gap of 1.39 eV. UV/vis absorption spectrum of **38** has a broad absorption between 500 and 800 nm with the lowest energy (*λ*_max_ = 676 nm) and the electrochemical energy gap is measured to be 1.58 eV.^[^ ^52b^ ^]^

**Figure 15:**
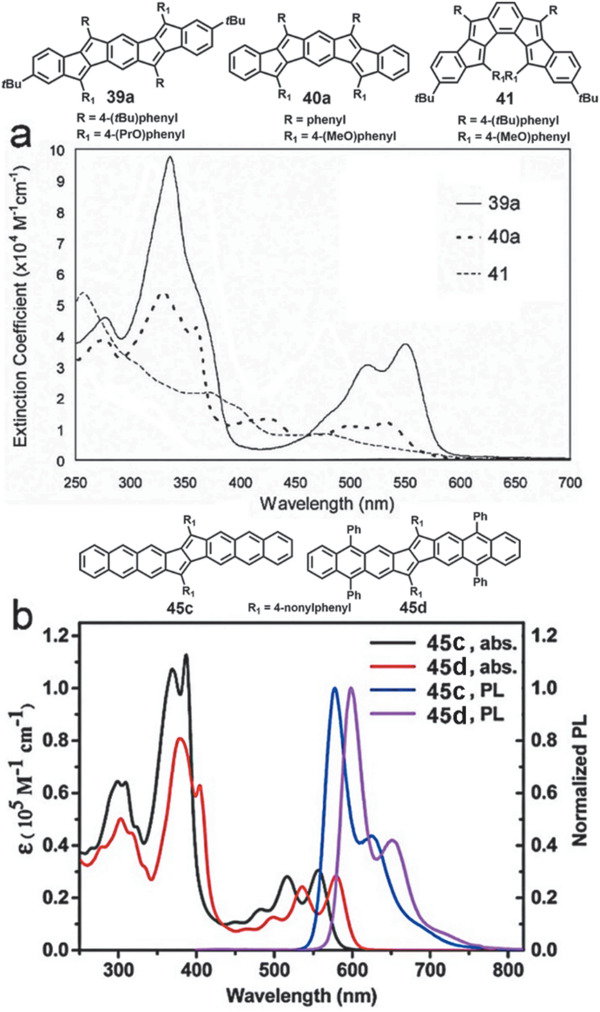
a) UV–vis spectra of **39a**, **40a**, and **41** in DCM. b) UV–vis and fluorescence spectra of **45c** and **45d**. Reproduced with permission.^[^ ^53^, ^58^ ^]^ Copyright 2010, American Chemical Society and Copyright 2015, Royal Chemical Society.

However, compared with indenofluorene‐based molecule **32 (** *λ*_max_ = 654 nm), as shown in **Figure** **15**a, all pentalene‐based compounds **39a**, **40a**, and **41** with different shapes exhibit significant blue‐shifting *λ*_max_ lower than 550 nm. Interestingly, the decreased lowest energy *λ*_max_ from 550 to 534 and 476 nm for **39a**, **40a**, and **41** indicates that the linear dipentalenes have the longest effective conjugation length.^[^ ^53^ ^]^ As shown in Figure 15b, compound **45c** shows a lowest energy *λ*_max_ at ≈560 nm, which is similar to those of **39a** and **43**, suggesting that the different units (e.g., benzene, naphthalene, and anthracene) as well as their positions (terminal or centered) fused onto the pentalene almost have no effect on their UV/vis absorption.^[^ ^53^, ^55^, ^58^ ^]^ For compounds **42a** and **42b**, their *λ*_max_ red‐shift toward 596 and 693 nm, respectively, which can be attributed to the absorption of the corresponding acenes with the pentalene attaching on them.^[^ ^54^ ^]^ Compounds **46a** and **46b** exhibit their most intense absorption peaks in the visible region centered at 756 and 786 nm, respectively, along with three bands in the near‐IR region up to 1700 nm.^[^ ^42e^ ^]^ Interestingly, for most five‐membered PCHs containing *s*‐indacene, pentalene, and indeno[1,2‐*b*]fluorene, they are considered as “dark” materials because of their none‐emissive character. However, indacene‐based molecule **34** show weak orange fluorescence with the emission *λ*_max_ at 664 nm and an extreme low quantum yield of ≈1% while pentalene‐based compound **45c** displays an obvious fluorescence with *λ*_max_ of 578 nm and a small Stokes shifts (549–685 cm^−1^) as shown in Figures 14b and 15b.^[^ ^52^, ^58^ ^]^ It is proposed that a low‐barrier potential energy surface crossing of S_0_ and S_1_ states (i.e., a conical intersection) will afford an efficient nonradiative pathway for fluorescence quenching.^[^ ^66^ ^]^ Therefore, as the outer benzenoid ring increases for **34** and **45c**, it is reasonable to assume that the barrier between S_0_ and S_1_ states must increase to such a level, where radiative decay is possible once again and thus their fluorescence restores. As a matter of fact, **34** has a much longer fluorescent lifetime of 1.4 ns, which is almost two orders of magnitude higher than the lifetime of indeno[1,2‐*b*]fluorene‐based compound (10–12 ps).

**Figure 16:**
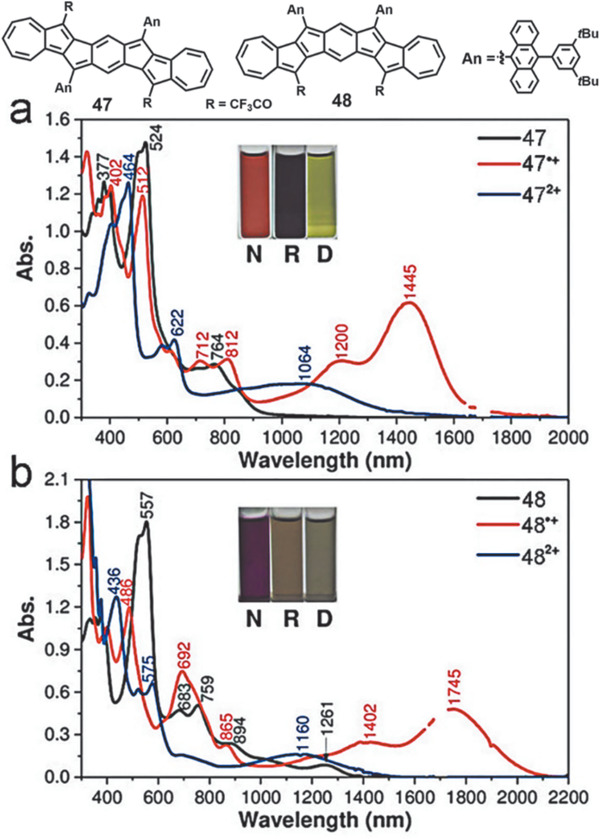
UV–vis–NIR absorption spectra of the neutral (N), radical cationic (R), and dicationic (D) states of a) **47** and b) **48** in DCM. Reproduced with permission.^[^ ^60^ ^]^ Copyright 2018, John Wiley & Sons.

As shown in **Figure** **16**a,b, compared with five‐membered PCHs, seven‐membered PCH **47** shows a much hypochromic shift with *λ*_max_ at 764 nm and *λ*_onset_ at 1170 nm after the incorporation of the strong electron‐withdrawing TFA groups.^[^ ^60^ ^]^ Compound **48** displays a much red‐shifted *λ*_max_ (1261 nm). The radical cations show long wavelength absorption *λ*_max_ at 1445 nm for **47** ^•+^ and 1745 nm for **48** ^•+^. The dications exhibit significantly blue‐shifted absorption compared with the corresponding radical cations, indicating the decreased lowest‐energy *λ*_max_ to 1064 and 1160 nm, respectively.

**Figure 17:**
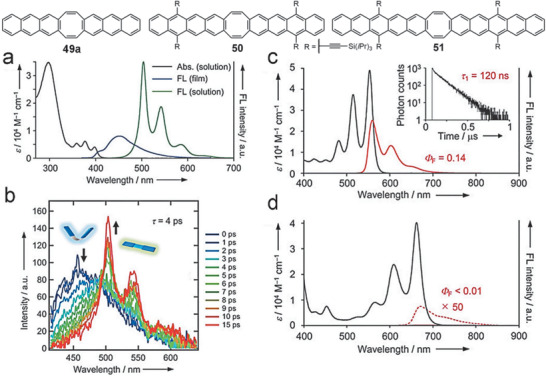
a) UV/vis and fluorescence spectra of **49a**. b) Time‐resolved fluorescence spectra of **49a** excited at 400 nm. UV/vis and fluorescence spectra of c) **50** and d) **51**. Excitation wavelengths are 515 nm for **50** and 609 nm for **51**. The fluorescence decay curve of **50** is shown in the inset of (c). Reproduced with permission.^[^ ^63b^ ^]^ Copyright 2018, John Wiley & Sons.

Interestingly, although PCHs with cyclooctatetraene (COT) ring contain two olefin functional groups, all the molecules **49–52** exhibit similar UV–vis absorption spectra with the trialkylsilylethyl‐substituted anthracene, tetracene, and pentacene as shown in **Figure** **17**.^[^ ^63^, ^64^, ^65^ ^]^ For example, **50** shows lowest energy absorption bands at 554 nm, slightly red‐shifted compared with that of tetracene derivative (534 nm).^[^ ^63^, ^67^ ^]^ Meanwhile, **51** and **52** exhibit the lowest energy *λ*_max_ at 662 and 648 nm, which are almost identical to that of the substituted pentacene (643 nm).^[^ ^63^, ^65^, ^68^ ^]^ Their electrochemical LUMOs and HOMOs do not vary significantly either. All these data imply that the additional two conjugated olefin groups brought slight change to their basic physicochemical properties. Nevertheless, **49a** shows the fluorescence with a large Stokes shift (5320 cm^−1^) in CH_2_Cl_2_ compared with those of **50** and **51** (160 cm^−1^), reflecting a fast V‐shaped‐to‐planar conformational change. Further transient absorption measurements explicate the different intramolecular singlet fission (SF) manners for **50** and **51**, namely, a fast and reversible SF, and a fast and quantitative SF, respectively.^[^ ^63b^ ^]^

**Figure 18:**
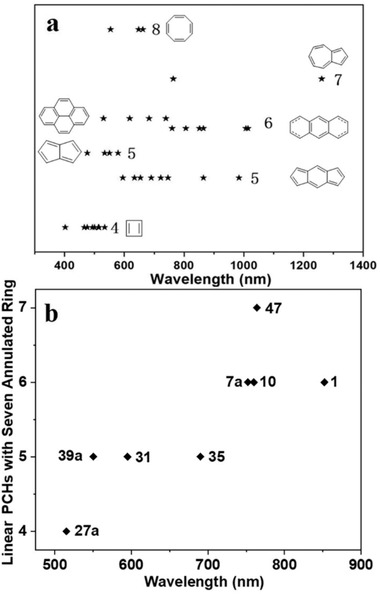
a) Lowest energy absorption *λ*_max_ versus all PCHs containing different membered ring. b) Lowest energy absorption *λ*_max_ versus linear PCHs with seven annulated rings containing different membered ring.

In this section, we mainly discuss the optoelectric properties of the PCHs containing four‐membered to eight‐membered ring. All the corresponding data are summarized in **Tables** **1** and **2**. In order to understand these data more comprehensively and clearly, we draw all their corresponding lowest energy absorptions *λ*_max_ together as shown in **Figure** **18**a. For PCHs containing four‐membered CBD and five‐membered pentalene unit, they exhibit lowest *λ*_max_ between 400 and 600 nm compared with all other PCHs. For PCHs containing COT unit, their *λ*_max_ is mainly dependent on the fused conjugated acene or arene on the two sides. For example, **51** has *λ*_max_ of 664 nm, which is mainly contributed from the pentacene backbone.^[^ ^63b^ ^]^ Interestingly, compared to PCHs containing pentalene, for PCHs containing other five‐membered part such as indacene, indenofluorene, etc., they display significantly red‐shifted *λ*_max_ between 600 and 800 nm, even reaching up to almost 1000 nm for **29b**,^[^ ^48b^ ^]^ on par with the *λ*_max_ (700–1000 nm) of the unsubstituted and substituted acenes higher than hexacene. Judged from this point, is it suggestive that PCHs containing indacene or indenofluorene will eventually exhibit similar and promising applicable characters as higher acenes? Actually, OFET performance of **32** has already reached to 7 cm^2^ V^−1^ s^−1^.^[^ ^69^ ^]^ Following this clue, higher PCHs containing azulene unit may also be a potential alternative for higher unsubstituted and substituted acene, considering their *λ*_max_ around 800–1200 nm.^[^ ^60^ ^]^ Nevertheless, the reported analogous compounds are rare and more congeners need to be synthesized and investigated to corroborate this assumption. Due to the twisted backbone of the acenes, the twistacene higher than heptacene only exhibits *λ*_max_ between 550 and 750 nm. To compared them exactly, we selectively take out several linearly fused PCHs with seven annulated rings containing four to seven‐membered ring and drawn as shown in Figure 18b. They generally show red‐shift absorption *λ*_max_ as the ring number increases from four to seven in PCHs when their molecular lengths fixed in seven‐fused rings. Through Figure 18a,b, we anticipate that a large picture of the lowest energy absorption *λ*_max_ for all the higher PCHs can be displayed and provide some guidance for future molecular design.

**Table 1:** Summary of the physicochemical properties and single crystal packing data of the PCHs with benzenoid ring ($E_{g}^{\mathrm{EC}}$: electrochemical energy gap; $E_{g}^{\mathrm{opt}}$: optical energy gap)

Entry	*λ*_onset_	*λ*_max_	$E_{g}^{\mathrm{opt}}$ ^a)^	$E_{g}^{\mathrm{EC}}$ ^b)^	LUMO^c)^	HOMO	Packing model	Intermolecular interaction	Ref.
	[nm]	[nm]	[eV]	[eV]	[eV]	[eV]			
1	912	852	1.36	1.30					^[^ ^5^ ^]^
3a	917		1.35	1.38	−3.50	−4.88	Herringbone packing	C–H–π (2.62 Å)	^[^ ^14^ ^]^
4b	905	865	1.37						^[^ ^16^ ^]^
5	1107		1.12						^[^ ^6a^ ^]^
6a	1033	1014	1.20	1.19			2D π‐stacked motif	π–π (3.38 Å)	^[^ ^6b^ ^]^
7a–c	775	752	1.60		−3.46	−5.06			^[^ ^18b^ ^]^
7d		958			−3.61		1D π‐stacked motif	π–π	^[^ ^7c^ ^]^
8	827	672	1.50	1.13	−3.60	−4.73			^[^ ^19c^ ^]^
9	496	445	2.50	2.54	−2.61	−5.15	2D π‐stacked motif	π–π (3.31 Å)	^[^ ^20a^ ^]^
15a		530	2.34^d)^	2.50	−2.10	−4.70	Herringbone packing	CH–π (2.79 Å)	^[^ ^30a^ ^]^
15b		618	2.01^d)^	2.09	−2.97	−5.06			^[^ ^30b,c^ ^]^
15c	611	577	2.03		−3.87	−5.90			^[^ ^30d^ ^]^
16						−5.10	1D π‐stacked motif	π–π (3.90 Å)	^[^ ^31^ ^]^
17		683	1.82^d)^	1.92			Herringbone packing	π–π (3.24 Å)	^[^ ^32^ ^]^
18		739	1.68^d)^	1.72			Herringbone packing	π–π (3.44 Å)	^[^ ^7b^ ^]^
19	550		2.25		−2.70	−4.95	Herringbone packing	CH‐π(≈2.40 and 2.60 Å)	^[^ ^33^ ^]^

a) $E_{g}^{\mathrm{opt}}$ = 1240/*λ*_onset_;

b) $E_{g}^{\mathrm{EC}}$ = LUMO–HOMO;

c) LUMO = HOMO + $E_{g}^{\mathrm{opt}}$;

d) $E_{g}^{\mathrm{opt}}$ = 1240/*λ*_max_.

**Table 2:** Summary of the physicochemical properties and single crystal packing data of the PCHs with nonbenzenoid ring ($E_{g}^{\mathrm{EC}}$: electrochemical energy gap; $E_{g}^{\mathrm{opt}}$: optical energy gap)

Entry	*λ*_onset_	*λ*_max_	$E_{g}^{\mathrm{opt}}$ ^a)^	$E_{g}^{\mathrm{EC}}$ ^b)^	LUMO^c)^	HOMO	Packing model	Intermolecular interaction	Ref.
	[nm]	[nm]	[eV]	[eV]	[eV]	[eV]			
21a	510	500	2.43		−2.96	−5.39	Herringbone packing	CH–π (4.19 Å)	^[^ ^9a^ ^]^
21b	500	491	2.48		−3.39	−5.87	Herringbone packing	π–π (3.47 Å)	^[^ ^36^ ^]^
21c	500	493	2.48		−3.42	−5.90	1D π‐stacked motif	π–π (3.47 Å)	
21d	523	513	2.37		−2.87	−5.24			^[^ ^9a^ ^]^
22b	470	466	2.64		−3.19	−5.83	2D π‐stacked motif	π–π (3.54 and 3.67 Å)	^[^ ^38^ ^]^
24	484	476	2.56		−2.91	−5.47			^[^ ^37a^ ^]^
25	550	476	2.25		−3.02	−5.28			
26	489	402	2.54		−2.94	−5.48			
27a	534	515	2.32	2.70	−2.96	−5.66	1D π‐stacked motif	π–π (3.38 Å)	^[^ ^37b^ ^]^
27b	534	515	2.32	2.76	−3.05	−5.81			
27c		534		2.53	−2.88	−5.41			
28a		746	1.66	1.10			1D π‐stacked motif	π–π (3.14 Å)	^[^ ^46^ ^]^
28b		756	1.64	1.20			1D π‐stacked motif	π–π (3.23 Å)	^[^ ^47^ ^]^
28c		865	1.43	1.04			Herringbone packing	CH–π (2.72 Å)	
29a							1D π‐stacked motif	π–π (2.73 Å)	^[^ ^48b^ ^]^
29b		984	1.26	0.98			1D π‐stacked motif	π–π (3.16 Å)	
31	633	595	1.95	1.75	−3.83	−5.58	2D π‐stacked motif	π–π (3.56 Å)	^[^ ^50^ ^]^
32	667	654	1.86	1.57	−4.11	−5.68	2D π‐stacked motif	π–π (2.85 Å)	
33	648	634	1.91	1.71	−3.95	−5.66	1D π‐stacked motif	π–π (3.36 Å)	
35	900	690	1.37	1.45	−3.82	−5.27	1D π‐stacked motif	π–π (3.33 Å)	^[^ ^51^ ^]^
36	1050	600	1.18	1.39			Herringbone packing	CH–π (2.80 Å)	^[^ ^52a^ ^]^
37		722	1.39	1.37	−3.88	−5.25	1D π‐stacked motif	π–π (3.59 Å)	^[^ ^52c^ ^]^
38		676	1.84	1.58	−3.44	−5.02	Herringbone packing	π–π (3.37 Å)	^[^ ^52b^ ^]^
42a		596	2.08^d)^	2.31	−3.04	−5.35	π‐stacked motif	π–π (3.4‐3.6 Å)	^[^ ^54^ ^]^
42b		693	1.79^d)^	1.94	−3.17	−5.11	π‐stacked motif	π–π (3.4‐3.6 Å)	
43		548	2.28				1D π‐stacked motif	π–π (3.31 Å)	^[^ ^55a^ ^]^
45c	582	556	2.13	2.07	−2.96	−5.03	1D π‐stacked motif	π–π (3.37 Å)	^[^ ^58^ ^]^
								CH–π (2.89 Å)	
45d	602	579	2.06	2.01	−3.02	−5.03	Herringbone packing	CH–π (2.78 to 2.84 Å)	
47	1170	764	1.06	1.31	−3.62	−4.93	Herringbone packing	CF–π (3.22 and 3.11 Å)	^[^ ^60^ ^]^
48	1442	1261	0.86	0.94	−3.88	−4.82	2D π‐stacked motif	π–π (3.79 Å) CH–π (2.83 Å)	^[^ ^60^ ^]^
52		648	1.91	2.40	−5.54	−3.14			^[^ ^65^ ^]^

a) $E_{g}^{\mathrm{opt}}$ = 1240/*λ*_onset_

b) $E_{g}^{\mathrm{EC}}$ = LUMO–HOMO

c) LUMO = HOMO + $E_{g}^{\mathrm{opt}}$

d) $E_{g}^{\mathrm{opt}}$ = 1240/*λ*_max_.

### 3.2 Antiaromaticity

PCHs with fused six‐membered benzenoid exhibit diatropic ring current, which can be considered to possess aromatic characteristic. On the contrary, PCHs with nonbenzenoid ring, such as CBD, indenofluorene and pentalene unit, will sustain paratropic ring currents that contribute to the antiaromaticity of these compounds, generally due to their prevalent quinoidal structures. To probe and differentiate the aromaticity and antiaromaticity, in 1996, a computational method nucleus independent chemical shift (NICS) has been utilized to determine the induced magnetic field under an external magnetic field.^[^ ^70^ ^]^ Negative and positive NICS values denote the aromatic rings and antiaromatic rings, respectively. However, NICS calculations will be restricted when applied in a multiring system that consists of more than one induced current circuit because the NICS value may represent the sum of many induced magnetic fields, which will lead to confused assignments of the aromaticity or antiaromaticity. Hence, one more powerful NICS methodology NICS‐XY‐Scan was developed later.^[^ ^71^ ^]^ This method can explore local, semiglobal, and global ring currents as well as the type of current(s) (diatropic vs paratropic) within a particular conjugated multiple‐ring system. This π‐only model removes the contribution of the *σ* electrons from the NICS values, affording ring current data produced solely from π‐electrons.^[^ ^1^, ^72^ ^]^

**Figure 19:**
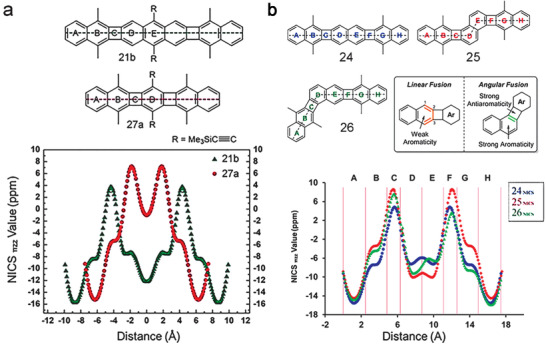
a) NICS‐XY scans of **21b** and **27a**. b) NICS‐XY scans of **24**, **25,** and **26**. Adapted with permission.^[^ ^37a,b^ ^]^ Copyright 2017, American Chemical Society and Copyright 2019, John Wiley & Sons.

As shown in **Figure** **19**a,b, the positive peak NICS values in the four‐membered CBD (C rings) increase slightly from 4.0 to 5.0 and 7.6 ppm for linear **21b**, **24**, and **27a**, suggesting the strongest paratropicity of **27a** as the central conjugated unit changing from anthracene to naphthalene and benzene.^[^ ^37a,b^ ^]^ This is further supported by the higher NICS value (around −6 ppm), corresponding to the outer CBD‐fused benzenoids (B and D rings) for **27a** compared with those of other two molecules (−7 ppm for **24** and −8 ppm for **21b**). Strikingly, the central benzenoid ring D in **27a** is so strongly dearomatized with a peak NICS value of only −1.0 ppm, indicating its nearly nonaromatic character. The terminal benzenoid A rings are not affected and retained high diatropicity with negative peak NICS values (around −14 ppm). Similar to compounds **27a**, **24**, and **21b**, compounds **28a**, **28c**, and **29a** also exhibit the decreased NICS value from 5.0 to 0.4 ppm and −0.8 ppm as the core conjugated linker changed from anthracene to naphthalene and benzene.^[^ ^48b^ ^]^ As shown in Figure 19b, compared with isomer **24**, both isomers **25** and **26** exhibit stronger paratropicity in the CBD rings and stronger diatropicity in the central naphthalenoid at the same time.^[^ ^37a^ ^]^ Therefore, linear fusion would reinforce the innate bond alternation in naphthalene, thus leading to a higher degree of dearomatization. On the contrary, angular fusion would weaken the bond alternation in naphthalene, thus resulting in a higher degree of aromaticity. This phenomenon also happens in the isomers of **31**, **32**, and **33** containing five‐membered indenofluorene.^[^ ^50^ ^]^

**Figure 20:**
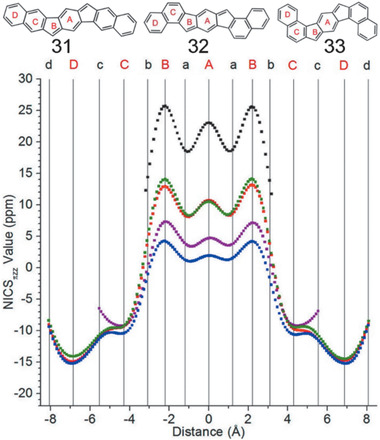
a) NICS‐XY scans of **31** (blue), **32** (red), **33** (green), *s*‐Indacene (black), and Indeno[1,2‐*b*]fluorene (purple). Reproduced with permission.^[^ ^50^ ^]^ Copyright 2016, American Chemical Society.

**Figure 21:**
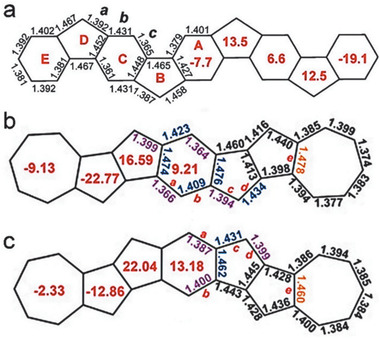
Selected bond lengths and NICS(1)ZZ value of a) **37**, b) **47,** and c) **48**. Reproduced with permission.^[^ ^52^, ^60^ ^]^ Copyright 2017, American Chemical Society and Copyright 2018, John Wiley & Sons.

As shown in **Figure** **20**, NICS‐XY measurements show that both angular **32** and **33** exhibit stronger antiaromaticity than linear **31** within the indacene core fused to naphthalene, in consideration of their much higher positive peak NICS values of 13 and 14 than 4 ppm for **31**.^[^ ^50^ ^]^ Fusion of the indacene core on the 2,3‐bond of anthracene gives the least antiaromatic indeno[1,2‐*b*]fluorene derivative **34** with a peak NICS‐XY scan value of 2 ppm and the minimum of zero ppm in the indacene core.^[^ ^52d^ ^]^ Diindenoanthracene **35** also exhibits very low NICS peak value around 1 ppm for the five‐membered ring area.^[^ ^51^ ^]^ As shown in **Figure** **21**a, NICX calculations of **37** provide the similar NICS values (12.5 and 13.5 ppm) to those of **32** and **33** with the two five‐membered rings, suggesting that **37** can be regarded as an antiaromatic hydrocarbon, where two antiaromatic indenofluorene units are annealed together via a weak aromatic benzene ring.^[^ ^52c^ ^]^ Compound **38** shows a slightly higher NICS value of 5.5 ppm than that of **31** (4 ppm).^[^ ^52b^ ^]^ For PCHs containing pentalene units, Haley group also studied antiaromaticity properties of DBP, DNP, and DAP through NICX‐XY scan method.^[^ ^50^ ^]^ The peak NICS_πZZ_ value decreases from around 12 to 7 and 4 ppm gradually, corresponding well with the peak NICS value (≈14 ppm) of a bispentalene derivative, where two pentalenes fuse onto a central benzene.^[^ ^73^ ^]^

As shown in Figure 21b,c, compounds **47** and **48** exhibited negative NICS values of −9.13 and −2.33 ppm in azulene ring, and 9.21 and 13.18 ppm in the central *s*‐indacene ring, indicating that these two molecules possessed an aromatic azulene‐fused antiaromatic *s*‐indacene structure.^[^ ^60^ ^]^

**Figure 22:**
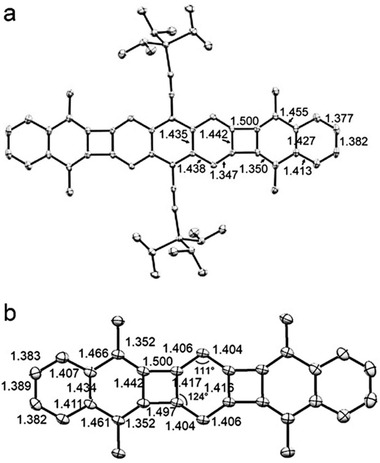
X‐ray crystal structure of a) **21b** and b) **27a** with highlighted bond lengths. Reproduced with permission.^[^ ^36^, ^37^ ^]^ Copyright 2017, American Chemical Society and Copyright 2018, John Wiley & Sons.

The antiaromaticity of nonbenzenoid PCHs can be further supported by the bond length study through the X‐ray crystallography. For **21b**, as shown in **Figure** **22**a, the phenylene linkage has alternating bond lengths of ≈1.35 and ≈1.44 Å, respectively while the bonds linking the acenoid segments have even greater single‐bond character with a bond length of 1.50 Å, revealing the increased π‐bond localization in order to minimize the paratropicity of CBD. The peripheral and central benzenoid rings that are not directly fused onto CBD rings are less affected and have more delocalized π‐bonds around 1.38 Å for **21b**.^[^ ^36^, ^37^ ^]^ However, for **27a**, as shown in Figure 22b, the central benzenoid bond lengths are almost identical with each other, slightly varying between 1.40 and 1.42 Å.^[^ ^37b^ ^]^ Similar with isomers **31**, **32**, and **33**, as shown in Figure 21a, compound **37** has large bond variation from 1.365 to 1.431 Å for bonds *a*, *b*, and *c*, illustrating an alternate double‐bond and single‐bond character. This large bond alternation indicates a significant contribution of the closed‐shell resonance form to the ground state.^[^ ^52c^ ^]^

### 3.3 Biradical

Open‐shell PCHs that possess multiple unpaired electrons have attracted wide investigation due to their potential applications in organic electronics, molecular spintronics, nonlinear optics, and energy conversion/storage devices.^[^ ^40b^ ^]^ The interacting pair of electrons in open‐shell PCHs can be in a spin‐paired singlet state (singlet biradical) or a spin‐parallel triplet state (triplet biradical).^[^ ^40^, ^74^ ^]^ Due to double spin polarization, most biradical PCHs have singlet ground states and the following molecules mainly fall into this group.^[^ ^75^ ^]^ Concurrent with the existent unpaired electrons, this type of molecules generally display broad ^1^H NMR signals at room temperature, which will gradually become sharp peaks with well‐defined coupling when lowering the temperature. In addition, in the UV–vis–NIR absorption spectra, there will be red‐shifted weak shoulder absorption bands in the NIR region compared with the strongest *λ*_max_. Furthermore, single‐crystal X‐ray diffraction can also provide the information of their open‐shell or closed‐shell structure PCHs. Aside from utilizing these phenomena to preliminarily judge the open‐shell character of these PCHs, electron spin resonance (ESR) measurements were employed to confirm the existence of unpaired electrons. The intensity of the biradical resonance character within an overall ground state structure can be defined as the biradical character index (*y*), which was estimated from the Natural Orbital Occupation Number of the LUMO on the basis of CASSCF(2,2)/6‐31G calculations. Its value ranges from 0 (indicating a completely closed shell) to 1 (pure open shell). Superconducting quantum interfering device (SQUID) measurements on the powders were conducted to investigate the temperature‐dependent magnetic susceptibility behavior and to estimate the excitation energies from the singlet ground state to the lowest triplet excited state.

**Figure 23:**
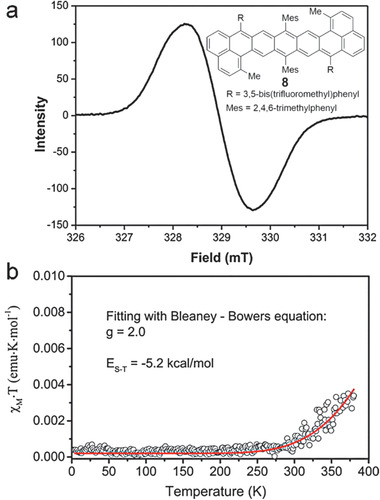
a) ESR spectrum of **8** in DCM recorded at −40 °C. b) *χT*−*T* plot for the solid sample of **8** in SQUID measurements. Reproduced with permission.^[^ ^19c^ ^]^ Copyright 2016, American Chemical Society.

It is predicted that larger acenes (higher than octacene) may possess open‐shell structures.^[^ ^76^ ^]^ For **6b**, at different measuring temperatures (room temperature and 115 K), all EPR experiments showed that there was an apparent signal, indicating the presence of a free radical at low concentration. However, the signal disappeared soon in the solution. Its ^1^H NMR spectra data could not be collected due to the fast decomposition in solution either. Compared with **6b**, nonazethrene **8** has progressively sharper peaks in ^1^H NMR, which appeared as the temperature decreased from 298 to 213 K, and the lower *λ*_max_ at 672 nm with two weak shoulder absorption bands at 800 and 895 nm, demonstrating its open‐shell singlet diradicaloids character.^[^ ^19c^ ^]^ Theoretical calculations indicates **8** possesses a moderate diradical character (*y* = 0.25) in singlet ground state. As shown in **Figure** **23**, both DCM solution and solid state of **8** displayed an apparent broad and strong ESR signal with a *g*_e_ value of 2.0, which is typical for largely delocalized singlet diradicaloids. SQUID measurement in Figure 23b showed that the *χT* product increases with the increase of temperature after 250 K, correlated to a thermal population from singlet to paramagnetic triplet state with a singlet–triplet energy gap (Δ*E*_S–T_) of −5.2 kcal mol^−1^. Further photostability tests revealed that **8** had the half‐life time of 16 h under ambient air and light condition, suggesting an improved biradical stability compared with **6b**.

As the bandgaps of **28b**, **28c,** and **29b** decreased, their amount of the singlet biradical characters (*y*) increased on the contrary from 30%, 50%, and 68%, respectively. Single crystals of these three compounds were also obtained, and the further analysis revealed that the length of the bonds connecting the phenalenyl parts and central aromatic units increased from 1.457 to 1.465 and 1.467 Å, which further affirms their increased singlet biradical character.^[^ ^48b^ ^]^

**Figure 24:**
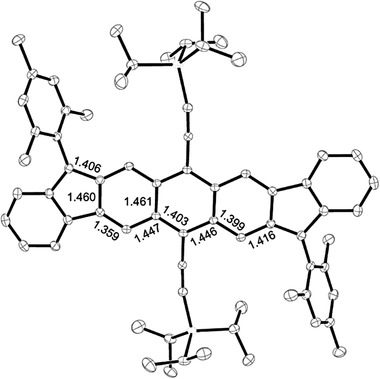
Solid‐state structure of **35** with selected bond distances (Å). Reproduced with permission.^[^ ^51^ ^]^ Copyright 2016, Springer Nature.

Compound **35** exhibited a strong *λ*_max_ at 690 nm with a weak shoulder that extended to 900 nm in the near infrared because of the symmetry‐forbidden S_0_→S_1_ transition. Compound **35** also had broadened ^1^H NMR peaks at 150°, which became sharp again at room temperature.^[^ ^51^ ^]^ As shown in **Figure** **24**, single‐crystal X‐ray diffraction analysis of **35** revealed that the bond lengths within the anthracene core vary greatly from 1.359 to 1.461 Å, which can be considered as a quinoidal unit including alternant double‐bonds and single‐bonds, demonstrating the antiaromaticity of **35**.^[^ ^51^ ^]^ Generally speaking, bond distances from the apical sp2 carbon to the central benzenoid in the reported mes‐substituted indenofluorene isomers range from 1.377 Å (closed shell) to 1.437 Å (open shell).^[^ ^77^ ^]^ Bond lengths of **35** (1.406 Å) and **36** (1.408 Å) from the apical sp2 carbon to the anthracene core fell between this range, indicating that the contribution is from both the pure open‐shell and closed‐shell structures for these two molecules.^[^ ^51^, ^52^ ^]^ It is worth to mention that **35** exhibited moderate biradical character of 0.62 with a remarkable stability, even in the presence of oxygen and at elevated temperatures.

Compound **46a** also had characteristic broad ^1^H NMR signals, suggesting its paramagnetic character.^[^ ^42e^ ^]^ In addition, the signals remained stable (slightly sharper) even at −90 °C, indicating appreciable triplet character. ESR measurement exhibited the signals without obvious spin–spin coupling, probably due to the long spin–spin distances and no intermolecular stacking. From the result of SQUID measurement, singlet–triplet energy gap (Δ*E*_S–T_) could be estimated to 3.4 kcal mol^−1^. Compound **48** with seven‐membered azulene unit also exhibited partially broadened ^1^H NMR peaks and two weak shoulder peaks at 894 and 1261 nm, contributed from (H, H) to (L, L) double excitation for the open‐shell diradicaloids,^[^ ^78^ ^]^ which were further confirmed by the strong ESR signal in solution both at room temperature and low temperature. As shown in Figure 21b, the bond lengths of *a* (1.364/1.366 Å) and *c* (1.399/1.394 Å) are longer than those in typical olefins (1.33–1.34 Å), and the bond b (1.409 Å) is much shorter than a typical single bond, indicating that the quinoidal character of the central *p*‐quinodimethane unit is diminished by the emergence of the diradical contribution.

From the above discussion, it seems that the large spatial overlap between HOMO and LUMO as well as small energy gap are responsible for the singlet biradical character. In addition, the stability of intrinsic reactivity in radical PCHs can be significantly improved through the delocalization of the spin‐over multiple conjugated backbones (e.g., zethrene^[^ ^19c^ ^]^ or phenalenyl^[^ ^48b^ ^]^) or the introduction of bulky groups (e.g., 3,5‐bis(trifluoromethyl)phenyl^[^ ^6b^ ^]^ or mesityl^[^ ^51^ ^]^).

## 4 Single Crystal Packing and OFET Performance

**Figure 25:**
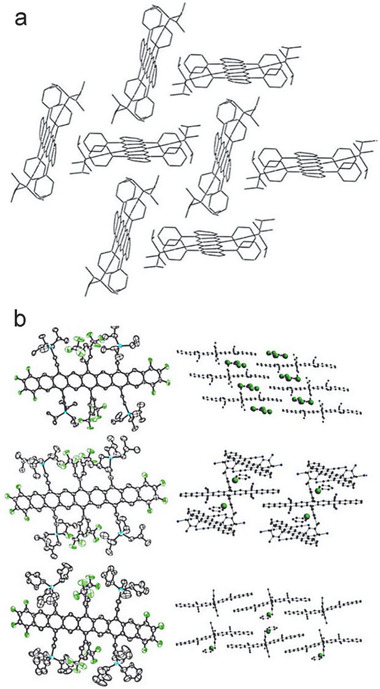
a) Herringbone packing motif of **3a**. b) Thermal ellipsoid plots for **6a** (top), **6b** (center), and **6c** (bottom), and packing diagrams. Reproduced with permission.^[^ ^6^, ^14^ ^]^ Copyright 2011 and 2008, John Wiley & Sons.

The molecular packing modes in single crystal usually play a vital role on determining their application performance.^[^ ^79^ ^]^ In this section, the single crystal packing modes of PCHs with different membered rings will be discussed. As shown in **Figure** **25**a, single crystal of **3a** exhibits edge‐to‐face herringbone packing in solid state, however, there are no π–π interactions between acene backbones.^[^ ^14^ ^]^ As shown in Figure 25b, due to the inserted solvent molecules, **6a** with triisopropylsilyl groups was slightly twisted and adopted a 2D π‐stacked motif with minimal π overlap through the entire space between the conjugated backbones of nonacene chromophores. Compound **6b** was almost perfect planar and exhibited only limited π–π overlapping, contributed from the terminal rings of the acenes. The adjacent backbones of nonacene packed almost perpendicularly to each other as 1D “slipped” stacks. Compared with **6a** and **6b**, **6c** was highly distorted and adopted a slipped 1D π‐stacking motif with much more overlap between the nonacene chromophores.^[^ ^6b^ ^]^ Single crystal of **9** packed in a 2D brickwork‐type fashion with the π–π distance of 3.31 Å, which is beneficial for an improved hole eletron mobility (up to 0.08 cm^2^ V^−1^ s^−1^).^[^ ^20a^ ^]^ Although single crystals of the unsubstituted hexacene have been found to possess a herringbone packing (face‐to‐edge) without π–π overlap (face‐to‐face) between adjacent molecules,^[^ ^80^ ^]^ the single crystals of unsubstituted higher acenes from heptacene to undecacene have not been reported yet and their packing motif are unexploited as far as we know.

**Figure 26:**
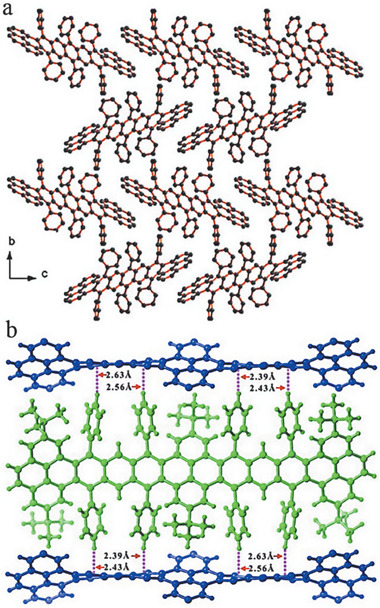
Single‐crystal packing modes of a) **18** and b) **19**.

All compounds **15a**, **17**, and **18** have similar packing pattern with minimal π–π interactions between the adjacent pyrene‐pyrene units as shown in **Figure** **26**a.^[^ ^7^, ^30^, ^32^ ^]^ Compared with **18**, the double‐sized molecule **19** formed the similar packing mode with **15a** but without any π–π interactions between two neighboring molecules.^[^ ^33^ ^]^ The strong twisted structure is due to the steric hindrance arising from the bulky substituents around the main skeleton. As shown in Figure 26b, strong intermolecular CH–π interactions were observed in this molecule packing and played a key role to account for this type of stacking arrangement. Compound **16** had a perfect 1D cofacial π–π packing mode with the π–π distance of 3.898 Å and exhibited an average mobility of 0.32 cm^2^ V^−1^ s^−1^.^[^ ^31^ ^]^

**Figure 27:**
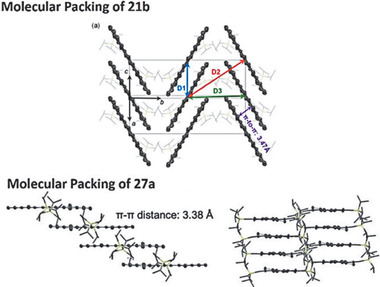
Crystal packing patterns of **21b** and **27a**. Reproduced with permission.^[^ ^37^, ^38^ ^]^ Copyright 2019 John Wiley & Sons and Copyright 2019, American Chemical Society.

Maybe due to the steric bulk effect of side phenyl groups, **21a** formed a face‐to‐face 1D packing mode with a relatively large intermolecular distance of 7.80 Å. There is a shorter edge‐to‐face interaction (4.19 Å) in **21a** between the phenyl substituents of opposing stacks.^[^ ^9a^ ^]^ As shown in **Figure** **27**a, after replacing the phenyl groups with a smaller methyl group, **21b** displayed the herringbone packing mode in two sets of columns with a close π–π interaction of 3.47 Å.^[^ ^38^ ^]^ As to **22a–c** without ethynyl groups, their crystal packing pattern transformed into 1D π‐stacking or 2D π‐stacking mode.^[^ ^38^ ^]^ As shown in Figure 27b, after the central part changed from anthracene to benzene, compound **27a** also stacked in a 1D column mode with the close π–π interaction of 3.38 Å.^[^ ^37b^ ^]^ The OFET performances of **21b–c** and **22a–c** were systematically studied by Miao group,^[^ ^38^ ^]^ and **22b** exhibited the best mobility of 2.9 cm^2^ V^−1^ s^−1^, which was higher than other molecules (**21b–c**, **22a,** and **22c**) by 1–3 magnitude. The authors attributed the high mobility to the favorable edge‐on orientation of molecules on the dielectric surface and the 2D nature of charge transport through X‐ray diffraction and AFM measurement. Xia group also observed similar phenomenon in studying the mobility of compound **27a**.^[^ ^37b^ ^]^ It is worth to mention that **27a‐TIBS** containing triisobutylsilyl (TIBS) formed a 2D “brick‐layer” packing mode while **27a** with TIPS group possessed a 1D column packing pattern. Although **27a** showed a high mobility of 0.19 cm^2^ V^−1^ s^−1^, the **27a‐TIBS** displayed the best mobility up to 0.52 cm^2^ V^−1^ s^−1^.

Unfortunately, isomers **24–26** containing TIPS–ethynyl substituents could not form the suitable crystals for X‐ray diffraction analysis. When replacing the TIPS–ethynyl group with the xylyl group, suitable single crystals of **24‐xylyl** formed and displayed a bow‐shaped structure with significant curvature in its π‐plane.^[^ ^37a^ ^]^

**Figure 28:**
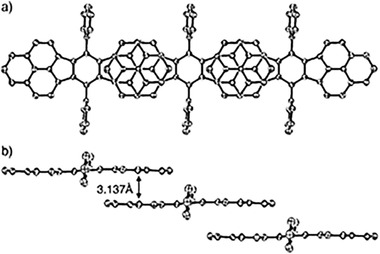
Crystal packing mode of **28a**. Reproduced with permission.^[^ ^46^ ^]^ Copyright 2005, John Wiley & Sons.

As shown in **Figure** **28**, **28a** formed a 1D π‐stacked motif with a slipped stacking arrangement, and π–π overlapping only appeared on the phenalenyl moieties. The average π–π distance was only 3.137 Å, which is substantially shorter than the van der Waals contact of carbon atoms (3.4 Å), indicating the large bandwidths in both HOMO and LUMO.^[^ ^46^ ^]^ The methyl‐substituted **28b** and core‐extended **29** also exhibited superimposed phenalenyl overlapping, which was identical to that of **28a**, except for the larger intermolecular π–π distance of 3.225 Å for **28b**.^[^ ^47^, ^48^ ^]^ The crystal structure of **28c** containing toluene molecules showed no obvious π–π interactions between the adjacent molecules.^[^ ^48a^ ^]^ Furthermore, single‐crystal X‐ray diffraction measurements indicated that both **31** and **32** formed 2D brickwork π‐stacked pattern while **33** adopted 1D π‐stacked motif.^[^ ^69^ ^]^ The influence of their shapes as well as the substituted groups on the performance of OFETs were systematically studied. Compound **31** (linear) exhibited an average hole mobility of 1.04 cm^2^ V^−1^ s^−1^ while anti **32** showed the mobility exceeding 7 cm^2^ V^−1^ s^−1^. Unfortunately, no device gave any OFET performance for syn **33**. Compound **35** exhibited the balanced ambipolar charge transport of 2 × 10^−3^ cm^2^ V^−1^ s^−1^ for hole mobility and 4 × 10^−3^ cm^2^ V^−1^ s^−1^ for electron mobility.^[^ ^51^ ^]^

For pentalene‐based molecules, **43** possesses a 1D π‐stacked motif. The effective offset stacking mode between the plane cores of the bispentalene core and a T‐shaped stacking motif between the peripheral phenyl rings were observed, which accounts for a hole mobility up to 0.25 cm^2^ V^−1^ s^−1^.^[^ ^55a^ ^]^ Wang group synthesized a series of diaceno[a,e]pentalenes including DBP, DNP, and DAP (**45b**), and studied their single crystal packing modes as well as their influence on the performance of single‐crystal FETs.^[^ ^57^ ^]^ When the stacking mode changed from an extremely‐weak sandwich‐herringbone arrangement for DBP to a relatively‐dense herringbone arrangement for DNP or a highly order 2D π–π arrangement with large overlap of the conjugated aromatic core for **45b**, their single‐crystal FET performance improved gradually from no performance for DBP to the higher hole mobility of 0.52 cm^2^ V^−1^ s^−1^ for DNP and 6.55 cm^2^ V^−1^ s^−1^ for **45b**.^[^ ^57^ ^]^ Chi group also synthesized two diaceno[a,e]pentalene molecules **45c** and **45d,** finding out that **45c** possessed a 2D brickwork π‐stacked motif with the π–π distance of 3.367 Å.^[^ ^58^ ^]^ However, there was no intermolecular π–π interaction between the DAP plane of **45d** due to the bulky substituents. Instead, there were multiple CH–π and π–π interactions between the pendant phenyl groups and the 4‐nonylphenyl as well as the DAP cores, which generated a closely 3D network. Chi group studied the OFET performance of **45c** and **45d** based on solution‐processed thin films. Compared with **45c** (0.001 cm^2^ V^−1^ s^−1^), **45d** exhibited much higher hole mobility up to 0.86 cm^2^ V^−1^ s^−1^, which is the highest mobility value obtained for solution‐processed pentalene‐based semiconductors.^[^ ^58^ ^]^ Different from the above‐discussed molecules **21b** and **27a‐TIBS** that form a 2D π‐stacked motif, there is no apparent π–π stacking between the main backbone for **45d**. The author attributed the improved mobility to the more closely packed structure in the solid state as well as good thin film morphology.^[^ ^58^ ^]^

## 5 Conclusion

In conclusion, we discussed the synthetic routes, basic physicochemical properties, single crystal packing modes, and the OFET applications of the linearly fused higher PCHs (*n* > 6). These PCHs are mainly classified into two groups in this review. 1) Acenes or π‐extended acene derivatives with only benzenoid rings; and 2) other oligoacene derivatives with four‐membered, five‐membered, seven‐membered, and eight‐membered rings. For the first group of PCHs only containing six‐membered rings, they generally exhibit aromaticity and the decreased stability when the backbone length increases gradually. Although stable single crystals for nonacene and dedecatwistarene have been obtained, their applications corresponding to photoelectric devices are rare, not to mention that the photogenerated or on‐surface generated unsubstituted higher acenes from heptacene to undecacene. Nevertheless, the second group of PCHs containing the membered‐rings different from six has significantly increased stability and at the same time, their antiaromaticity increases simultaneously. With this advantage, they exhibited excellent hole mobility up to 7 cm^2^ V^−1^ s^−1^.^[^ ^69^ ^]^

Nevertheless, there are still several challenges that need to be faced.

### 5.1 Developing More Novel Methods/Strategies to Approach the Higher PCHs

Through the above discussion, we know that Xia group synthesized a series of PCHs with four‐membered CBD ring^[^ ^36^, ^37^ ^]^ through a novel Pd‐catalyzed system, and Hashmi group developed a gold‐catalyzed reaction to form various pentalene or dipentalene‐based PCHs for realizing the aim to control the shape of the target molecules.^[^ ^42^, ^55^ ^]^ Nevertheless, it is apparent that the synthetic work to higher acenes is still tedious due to the limited method for the construction of the key acene precursor backbones. Recently, Hamura group investigated extensively on constructing various isobenzofuran‐based acene prepursors.^[^ ^81^ ^]^ Can his method be utilized to construct the higher acene precursors?

### 5.2 Obtaining More Novel PCHs

For PCHs containing benzenoid ring, as far as we know, the longest substituted acene is molecule **6a**.^[^ ^6b^ ^]^ Therefore, the potential properties of the higher substituted acenes than nonacene are still unknown. Hence, success in synthesizing and separating stable substituted decacene will be very interesting. It is worth to mention that although Chi group synthesized two seven‐fused linear PCHs based on azulene and investigated comprehensively their optoelectrical, antiaromatic and biradical properties, the reported analogues are still rare.^[^ ^60^ ^]^ Only with different sufficient novel PCHs in hand, we can further systematically study their properties and potential applications.

### 5.3 Exploiting More Bizarre Applications

For PCHs containing four‐membered CBD and five‐membered indenofluorene or pentalene unit, their OFET performance has been studied intensively recently. Nevertheless, although many higher PCHs with stable or unstable biradical character have been reported and their basic physicochemical properties have been elaborated, no big breakthrough seems to be seen in their applications. Thus, the distinct founding in this direction will be very interesting and encouraging.

## Conflict of Interest

The authors declare no conflict of interest.
